# MRI-enabled ferroptosis self-amplifying nanoplatform synergizes with photothermal therapy to enhance chemotherapeutic efficacy against pancreatic cancer

**DOI:** 10.1016/j.mtbio.2026.103469

**Published:** 2026-07-16

**Authors:** Pan Yang, Shuai He, Mingdong Xu, Yanying Sun, Jingyi Gao, Jian Chen, Tongtong Niu, Liguo Hao

**Affiliations:** Department of Molecular Imaging, School of Medical Technology, Qiqihar Medical University, Qiqihar, Heilongjiang, 161006, PR China

**Keywords:** Pancreatic cancer, Chemoresistance, Ferroptosis, Calcium overload, Photothermal therapy, MRI

## Abstract

Pancreatic cancer responds poorly to conventional chemotherapy, largely because of the pronounced resistance of tumor cells to chemotherapy-induced apoptosis. Ferroptosis, a non-apoptotic form of programmed cell death, has emerged as a promising strategy to overcome this resistance. However, its therapeutic efficacy is often limited by insufficient hydrogen peroxide (H_2_O_2_) and excessive glutathione (GSH) in the tumor microenvironment (TME). Herein, we developed a nanoplatform, HM-MnO_2_@DOX/CaO_2_@PDA/HA (HMDCPH), using hollow mesoporous manganese dioxide (HM-MnO_2_) as a carrier to co-deliver doxorubicin (DOX) and calcium peroxide (CaO_2_). The crosslinked PDA/HA shell enhanced both the tumor-targeting capability and biocompatibility of the nanoplatform. In the TME, HM-MnO_2_ depleted GSH and promoted reactive oxygen species (ROS) generation, whereas CaO_2_ decomposition generated H_2_O_2_ and released Ca^2+^, inducing mitochondrial calcium overload and further aggravating oxidative stress. These synergistic effects enhanced lipid peroxidation (LPO) and exacerbated ferroptosis-related oxidative damage. Moreover, the near-infrared (NIR)-triggered photothermal effect further strengthened the antitumor efficacy of HMDCPH. In addition, nanoplatform degradation released Mn^2+^, enabling T_1_-weighted magnetic resonance imaging (MRI). Collectively, this study presents a synergistic nanotherapeutic strategy that integrates chemotherapy, photothermal therapy, and ferroptosis-related mechanisms to overcome chemoresistance in pancreatic cancer.

## Highlights

• Targeted HMDCPH nanoplatform with MR imaging synergizes against pancreatic cancer.
• Induces ferroptosis-related oxidative damage via GSH depletion and enhanced ROS production.
• CaO_2_-released Ca^2+^ triggers mitochondrial Ca^2+^ overload, aggravating oxidative stress.
• NIR photothermal effect enhances antitumor efficacy.
• Overcomes chemoresistance by integrating chemo, photothermal, and ferroptosis.

## Graphical abstract

**Figure:**
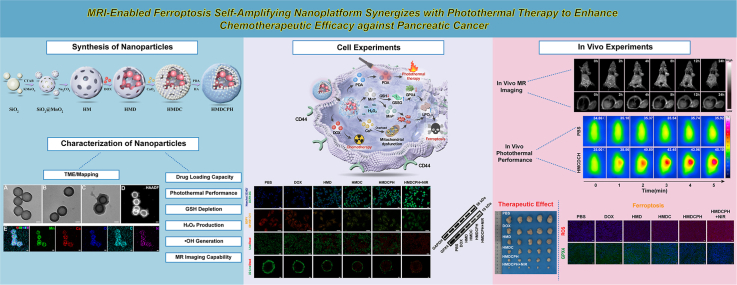

## 1 Introduction

Pancreatic cancer is one of the most aggressive solid malignancies and remains a formidable clinical challenge because of its poor five-year survival rate [1,2]. Current clinical treatment strategies still rely largely on apoptosis-inducing chemotherapeutic agents. However, intrinsic or treatment-acquired apoptosis resistance in tumor cells often limits chemotherapeutic efficacy [3]. Therefore, alternative non-apoptotic cell death pathways are urgently needed to enhance the elimination of resistant cancer cells [4]. Ferroptosis is a form of non-apoptotic cell death driven by lipid peroxidation (LPO). During this process, intracellular glutathione (GSH) depletion disrupts the glutathione peroxidase 4 (GPX4)-centered antioxidant defense system, whereas Fenton or Fenton-like reactions exacerbate oxidative stress, leading to reactive oxygen species (ROS) accumulation, lipid peroxide formation, membrane damage, and ultimately cell death [5,6]. Thus, ferroptosis-related oxidative damage provides a promising therapeutic approach for overcoming apoptosis resistance in pancreatic cancer.

Although hydrogen peroxide (H_2_O_2_) is more abundant in the tumor microenvironment (TME; 10–100 μM) than in normal tissues, its concentration remains insufficient to induce lethal ROS generation [7,8]. In addition, elevated GSH levels and related antioxidant defense systems in tumor cells enable rapid ROS scavenging, thereby markedly compromising ferroptosis induction efficiency [9,10]. Therefore, simultaneous local H_2_O_2_ supplementation and GSH depletion are crucial for improving ferroptosis-based therapy [11,12]. Previous strategies based on liposomes or polymersomes for direct H_2_O_2_ delivery have been limited by poor stability, premature leakage, and off-target toxicity [13,14]. Consequently, metal peroxides with TME-responsive H_2_O_2_-generating properties have attracted increasing attention [15,16]. Among them, calcium peroxide (CaO_2_) can react in the acidic TME to generate H_2_O_2_, thereby disrupting redox homeostasis. Meanwhile, the released Ca^2+^ induces intracellular calcium overload and subsequent mitochondrial damage, further exacerbating oxidative stress [17,18]. However, CaO_2_ nanoparticles (NPs) exhibit inherent instability in the circulatory system and are prone to rapid degradation in vivo, restricting their accumulation at tumor sites and limiting effective H_2_O_2_ replenishment. Therefore, suitable nanocarriers are essential for preserving the stability of CaO_2_ NPs and promoting their efficient tumor accumulation.

Owing to its high specific surface area, porous structure, and favorable biodegradability, hollow mesoporous manganese dioxide (HM-MnO_2_, HM) has emerged as an attractive nanocarrier for loading drugs, proteins, and small nanoparticles [19,20]. In addition to serving as a nanocarrier, HM-MnO_2_ can deplete intracellular GSH and release Mn^2+^ under TME-relevant conditions, thereby disrupting redox homeostasis and further enhancing oxidative stress via Fenton-like catalytic activity, ultimately facilitating ferroptosis-related damage in tumor cells [21,22]. Meanwhile, the released Mn^2+^ can serve as a T_1_-weighted magnetic resonance imaging (MRI) contrast agent for tumor imaging [23].

Poor accumulation of anticancer drugs in tumors remains a key factor limiting therapeutic efficacy [24]. Although metal-based nanomedicines show promising therapeutic potential, their inadequate targeting ability often results in off-target toxicity in healthy tissues [25]. Hyaluronic acid (HA) is commonly used to improve tumor targeting because of its specific affinity for CD44 receptors, which are overexpressed in many tumors [26]. HA coating can promote nanomedicine accumulation at tumor sites and improve biocompatibility [27]. Moreover, polydopamine (PDA) can act not only as a photothermal material but also as a pH-responsive gatekeeper for controlled drug release. Under physiological conditions, PDA forms a dense adhesive film on the nanocarrier surface, thereby preventing premature drug leakage during systemic circulation. In contrast, the acidic TME facilitates targeted drug release by promoting PDA degradation [28,29].

**Scheme 1:**
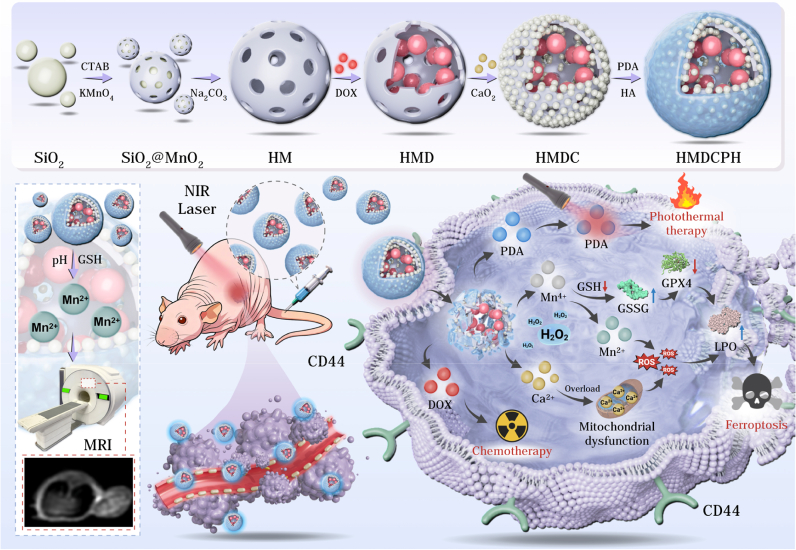
Diagrammatic representation of the construction strategy and antineoplastic mechanism of HM-MnO_2_@DOX/CaO_2_@PDA/HA (HMDCPH).

Based on these considerations, we developed a multifunctional nanoplatform, HM-MnO_2_@DOX/CaO_2_@PDA/HA (HMDCPH) (Scheme 1). In this system, HM-MnO_2_ serves as the core nanocarrier for the co-delivery of DOX and CaO_2_ and is coated with a crosslinked PDA/HA layer. HMDCPH integrates multiple functions: (1) HA enhances tumor targeting and cellular uptake via CD44 receptor-mediated recognition, while PDA confers acid-responsive drug release and photothermal conversion capabilities. (2) In the acidic and GSH-rich TME, HMDCPH undergoes responsive degradation to release DOX, Mn^2+^, Ca^2+^, and H_2_O_2_, accompanied by GSH depletion. (3) The released Mn^2+^ catalyzes Fenton-like reactions to generate hydroxyl radicals (·OH), which, together with GSH depletion, promotes ROS accumulation and enhances LPO, thereby aggravating ferroptosis-related oxidative damage. (4) In addition, mitochondrial dysfunction induced by intracellular Ca^2+^ overload, together with CaO_2_-derived H_2_O_2_, further aggravates oxidative damage in tumor cells. Upon near-infrared (NIR) laser irradiation, the photothermal effect further enhances the antitumor efficacy of HMDCPH. (5) Meanwhile, the released Mn^2+^ can serve as a T_1_-weighted MRI contrast agent for tumor imaging. Overall, HMDCPH addresses key barriers in pancreatic cancer nanotherapy, including poor tumor targeting, limited H_2_O_2_ availability, and strong antioxidant defenses. By integrating these complementary therapeutic mechanisms, HMDCPH provides a promising strategy for overcoming resistance to chemotherapy-induced apoptosis and thereby improving therapeutic efficacy against pancreatic cancer.

## 2 Results and discussion

### 2.1 Preparation and characterization

**Fig. 1:**
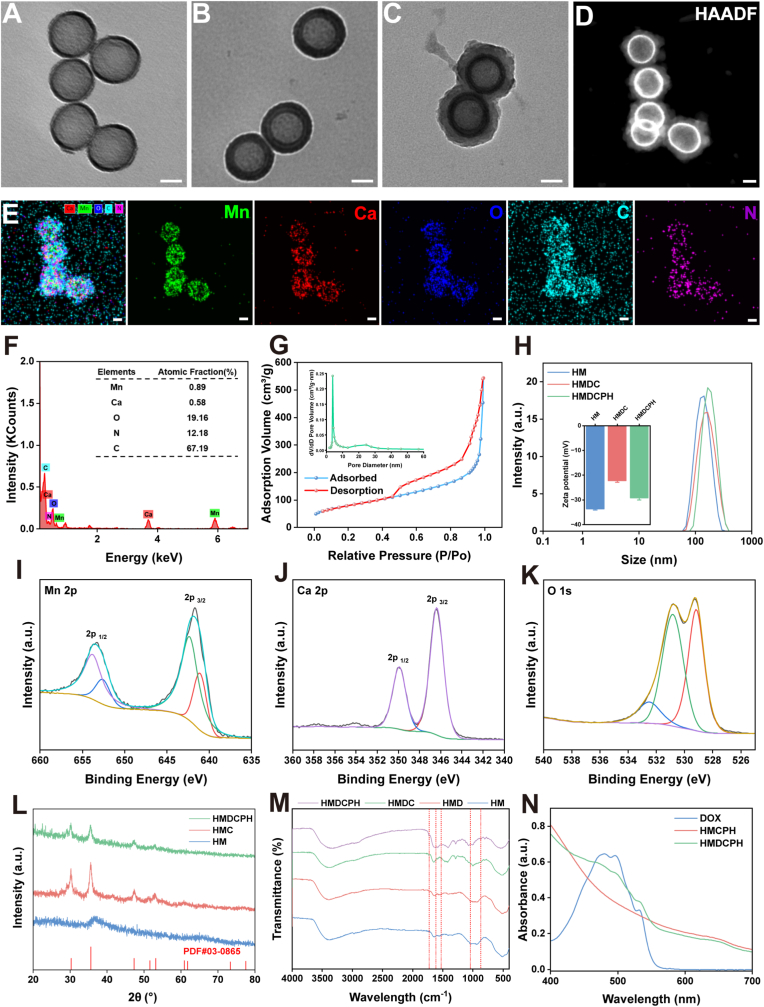
TEM images of HM (A), HMDC (B), and HMDCPH (C) (scale bar: 50 nm). HAADF-STEM image (D), corresponding elemental mapping images (E), and EDS spectrum (F) of HMDCPH NPs (scale bar: 50 nm). N_2_ adsorption–desorption isotherms and pore size distribution of HM (G). Hydrodynamic diameter and zeta potential of HM, HMDC, and HMDCPH (H). XPS spectra of Mn 2p (I), Ca 2p (J), and O 1s (K) for HMDCPH. XRD patterns (L), FT-IR spectra (M), and UV–vis spectra (N) of the different nanoparticles.

As illustrated in Scheme 1, HM-MnO_2_ (HM) was first synthesized using a hard-template method. DOX was subsequently introduced into the hollow mesoporous structure through solvent evaporation, after which CaO_2_ was deposited in situ. The resulting HM-MnO_2_@DOX/CaO_2_ (HMDC) was then coated with a crosslinked PDA/HA composite layer to yield HM-MnO_2_@DOX/CaO_2_@PDA/HA (HMDCPH). Transmission electron microscopy (TEM) revealed that HM possessed a uniform hollow structure (Fig. 1A). After DOX loading and CaO_2_ deposition, HMDC NPs exhibited a distinct double-layer structure (Fig. 1B and S1A). Following further coating with PDA/HA, an apparent outer shell was observed on the nanoparticle surface (Fig. 1C and S1B). High-angle annular dark-field scanning transmission electron microscopy (HAADF-STEM) imaging, elemental mapping, and energy-dispersive X-ray spectroscopy (EDS) confirmed the homogeneous distribution of Mn, Ca, O, N and C in HMDCPH NPs (Fig. 1D–F). The corresponding elemental contents were 0.89%, 0.58%, 19.16%, 12.18% and 67.19%, respectively, further supporting the successful synthesis of HMDCPH. In addition, the N_2_ adsorption–desorption isotherm exhibited typical reversible type IV characteristics, together with a high specific surface area of 288.73 m^2^ g^−1^ and an average pore diameter of 10.67 nm, confirming the well-defined hollow mesoporous structure of HM NPs (Fig. 1G), which is favorable for efficient DOX and CaO_2_ loading. Dynamic light scattering (DLS) analysis showed that the hydrodynamic diameter progressively enlarged during stepwise assembly, reaching 130.9 ± 1.0583, 149.4 ± 1.0440, and 168.0 ± 0.9539 nm for HM, HMDC, and HMDCPH NPs, respectively (Fig. 1H). The corresponding zeta potential changes revealed that the nanoparticle surface charge became less negative after DOX and CaO_2_ loading, but more negative after PDA/HA coating, confirming the successful construction of the nanoplatform. HMDCPH NPs maintained a stable hydrodynamic diameter in PBS and RPMI 1640 medium over a 7-day incubation period (Fig. S2), indicating excellent colloidal stability under physiological conditions.

X-ray photoelectron spectroscopy (XPS) confirmed the presence of Mn, Ca, and O in HMDCPH NPs (Fig. S3). In the Mn 2p spectrum, the characteristic Mn^4+^ peaks at 642.4 eV (2p_3/2_) and 653.9 eV (2p_1/2_) verified the formation of MnO_2_ (Fig. 1I), whereas the Ca 2p peaks at 346.4 eV (2p_3/2_) and 350.0 eV (2p_1/2_) confirmed the presence of Ca^2+^ (Fig. 1J). In addition, the O 1s spectrum showed three peaks at 529.2, 530.8, and 532.5 eV, with the peak at 532.5 eV attributed to peroxide ions (O_2_^2−^), verifying the successful deposition of CaO_2_ in HMDCPH NPs (Fig. 1K). X-ray diffraction (XRD) was used to further characterize the crystalline phases of the prepared nanomaterials. As shown in Fig. 1L, both HM-MnO_2_@CaO_2_ (HMC) and HMDCPH displayed characteristic diffraction peaks at 30.1°, 35.5°, 47.4°, and 52.9°, which matched well with the standard pattern of CaO_2_ (JCPDS No. 03-0865), further confirming the presence of CaO_2_ in the materials. Fourier transform infrared (FT-IR) spectroscopy was employed to verify the stepwise assembly process. The Mn–O stretching band at 521 cm^−1^ was characteristic of the MnO_2_ framework. The peak at 1730 cm^−1^ was ascribed to C=O stretching of the quinone ring, suggesting successful DOX loading. Meanwhile, the O–O bridge stretching vibration at 870 cm^−1^ demonstrated the presence of CaO_2_. In addition, the peaks at 1618 and 1527 cm^−1^ were assigned to the aromatic C=C stretching and secondary amine/imine N–H bending vibrations of PDA, respectively, whereas the C–O stretching peak at 1043 cm^−1^ originated from the sugar moieties of HA, supporting the successful formation of the PDA/HA coating (Fig. 1M and S4). Moreover, ultraviolet–visible (UV–vis) spectroscopy of HMDCPH revealed that although the absorbance of DOX at 485 nm slightly decreased after encapsulation, its characteristic absorption peak remained clearly visible, further confirming the successful loading of DOX into the HMDCPH nanoplatform (Fig. 1N).

### 2.2 In vitro photothermal performance

**Fig. 2:**
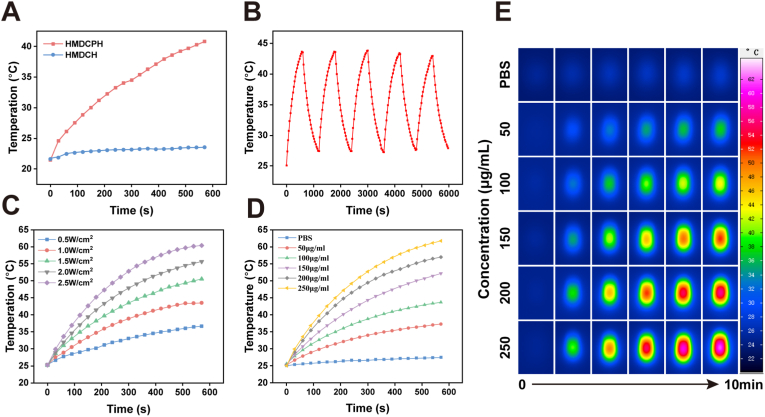
Time-temperature curves of HMDCH and HMDCPH at the same concentration under NIR irradiation at an identical power density (A). Photothermal cycling profile of the HMDCPH dispersion under repeated NIR irradiation (B). Time-temperature curves of HMDCPH under different NIR irradiation power densities (C). Time-temperature curves (D) and infrared thermal images (E) for PBS and HMDCPH dispersions of varying concentrations.

Given the photothermal properties of PDA, the photothermal conversion performance of HMDCPH NPs was systematically evaluated. Upon irradiation with an 808 nm NIR laser (1.0 W cm^−2^), HMDCPH exhibited a more pronounced temperature elevation than HM-MnO_2_@DOX/CaO_2_@HA (HMDCH) (Fig. 2A), likely because of PDA-mediated photothermal conversion, suggesting the potential of HMDCPH as a photothermal agent. Moreover, the HMDCPH dispersion maintained a stable maximum temperature over five irradiation–cooling cycles, confirming its excellent photothermal stability (Fig. 2B). The cooling curve analysis showed that HMDCPH achieved a photothermal conversion efficiency of 33.41% (Fig. S5). After 10 min of NIR irradiation at different power densities (0.5−2.5 W cm^−2^), the temperature of the HMDCPH dispersion (100 μg mL^−1^) increased from 36.66 to 60.39 ^°^C (Fig. 2C), demonstrating a power-dependent photothermal effect. Similarly, after 10 min of NIR irradiation at 1.0 W cm^−2^, HMDCPH dispersions at concentrations of 50−250 μg mL^−1^ reached 37.30−61.74 ^°^C, whereas PBS reached only 27.48 ^°^C (Fig. 2D), indicating a concentration-dependent photothermal effect. Consistently, real-time infrared thermal imaging revealed gradually enhanced thermal signal intensity with increasing irradiation time and nanoparticle concentration (Fig. 2E), further supporting the concentration-dependent photothermal behavior of HMDCPH.

### 2.3 Drug loading capacity and release behavior

**Fig. 3:**
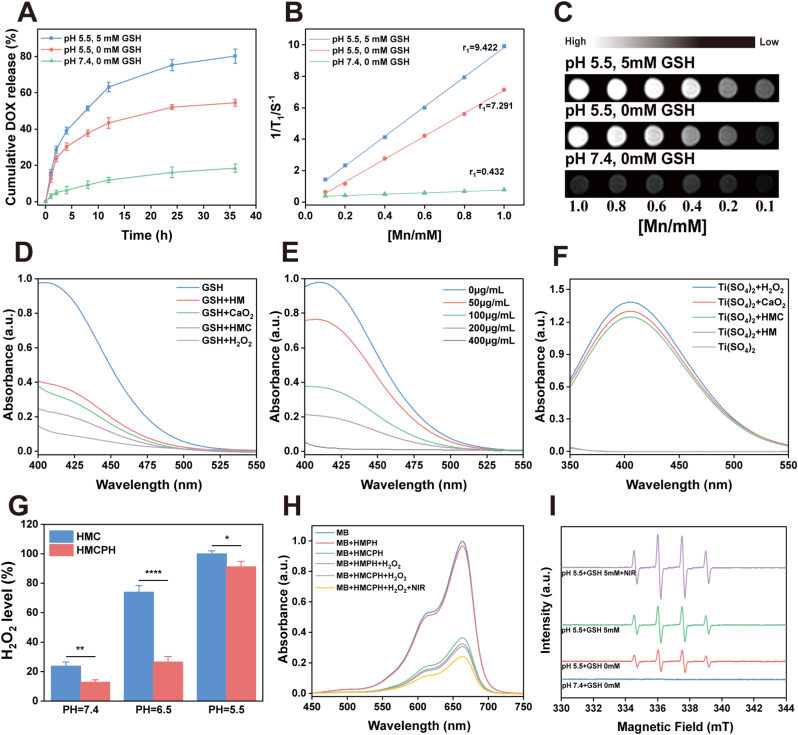
Drug release profiles (A), changes in r_1_ (B), and T_1_-weighted MR images (C) of HMDCPH under different conditions. UV–vis spectra for GSH depletion across treatment groups (D) and at varying HMCPH concentrations (E). UV–vis spectra for H_2_O_2_ generation across treatment groups (F). H_2_O_2_ levels generated by HMC and HMCPH under different pH conditions (G). UV–vis spectra for ·OH generation across treatment groups (H). ESR spectra of HMCPH NPs under different treatment conditions (I). ∗*p* < 0.05, ∗∗*p* < 0.01, ∗∗∗*p* < 0.001, and ∗∗∗∗*p* < 0.0001.

The HMDCPH platform leverages the hollow mesoporous structure of HM-MnO_2_ to achieve efficient drug loading, with a DOX loading capacity (DL) of 17.31% and an encapsulation efficiency (EE) of 69.88% (Fig. S6). The stimuli-responsive release behavior of DOX was investigated under varied pH and GSH conditions. Under neutral physiological conditions (pH 7.4, 0 mM GSH), only 18.45% of the loaded DOX was released, indicating that HMDCPH exhibited favorable stability during blood circulation. In contrast, the cumulative DOX release increased to 54.46% under acidic conditions (pH 5.5, 0 mM GSH), confirming the pH-triggered disassembly of HMDCPH. When the TME was further simulated (pH 5.5, 5 mM GSH), the cumulative DOX release reached 80.30% (Fig. 3A), which was primarily attributed to H^+^- and GSH-induced structural disruption of HMDCPH. These findings demonstrate that HMDCPH has the potential to minimize premature drug leakage under physiological conditions while enabling on-demand, enhanced DOX release in the TME through dual-responsive mechanisms.

### 2.4 In vitro MR imaging capability

To evaluate the MR imaging performance of HMDCPH NPs, T_1_-weighted MRI was performed using HMDCPH dispersions at various concentrations under different pH and GSH conditions. Under neutral physiological conditions (pH 7.4, 0 mM GSH), negligible signal enhancement was observed, with a low longitudinal relaxivity (r_1_) of approximately 0.432 mM^−1^ s^−1^. In contrast, the T_1_-weighted signal intensity markedly increased under acidic and reductive conditions (pH 5.5, 5 mM GSH), with the r_1_ value increasing to approximately 9.422 mM^−1^ s^−1^ (Fig. 3B and C). These results suggest that HMDCPH NPs undergo responsive degradation under TME-mimicking conditions, thereby releasing Mn^2+^ and serving as a T_1_-weighted contrast agent for tumor-targeted MR imaging.

### 2.5 In vitro GSH consumption and H_2_O_2_/·OH generation capacities

As a pivotal redox buffer, GSH can scavenge therapeutic ROS [30,31]. Therefore, GSH depletion can enhance ROS-mediated anticancer therapy. The GSH consumption capacity of the nanoplatform was determined using the DTNB (5,5′-dithiobis (2-nitrobenzoic acid)) assay. GSH levels were lower in the HM, CaO_2_, HM-MnO_2_@CaO_2_ (HMC), and H_2_O_2_ groups than in the control group (Fig. 3D). Furthermore, GSH levels progressively decreased with increasing concentrations of HM-MnO_2_@CaO_2_@PDA/HA (HMCPH) (Fig. 3E). These results suggest that GSH was efficiently consumed through redox reactions mediated by HM and by H_2_O_2_ generated from CaO_2_ hydrolysis.

The H_2_O_2_ generation capacity of the nanoplatform was evaluated using titanium sulfate [Ti(SO_4_)_2_]. Upon reaction with H_2_O_2_, the Ti(SO_4_)_2_ solution changed from colorless to yellow and exhibited a characteristic absorbance peak at 410 nm. The HMC, CaO_2_, and H_2_O_2_ groups exhibited pronounced absorbance peaks, whereas the HM group showed negligible absorbance (Fig. 3F), indicating H_2_O_2_ generation via CaO_2_ hydrolysis. The H_2_O_2_ release behavior of HMC and HMCPH NPs was further examined using an H_2_O_2_ detection kit at pH 7.4, 6.5, and 5.5. H_2_O_2_ release showed clear pH dependence, with greater release observed at lower pH values. Notably, HMC consistently released more H_2_O_2_ than HMCPH under identical conditions (Fig. 3G), suggesting that the PDA/HA shell reduced the exposure of CaO_2_ to the aqueous environment and helped prevent premature hydrolysis.

·OH generation was assessed using the methylene blue (MB) bleaching assay. Under TME-mimicking conditions, the HMCPH-treated group exhibited markedly lower MB absorbance than the HM-MnO_2_@PDA/HA (HMPH) group. Notably, the MB absorbance in the HMCPH-treated group was comparable to that in the HMPH + H_2_O_2_ group, indicating that H_2_O_2_ released from CaO_2_ could be efficiently converted into ·OH via Mn^2+^-mediated Fenton-like reactions. In addition, NIR irradiation enhanced MB bleaching (Fig. 3H), which may be attributed to photothermally induced local temperature elevation that facilitated the Mn^2+^-mediated catalytic reaction [32]. Electron spin resonance (ESR) spectroscopy was further employed to investigate nanoplatform-mediated ·OH generation under different conditions. Only weak ·OH signals were detected under physiological conditions (pH 7.4, 0 mM GSH). In contrast, strong ·OH signals emerged under TME-mimicking conditions (pH 5.5, 5 mM GSH), demonstrating that the nanoplatform could efficiently generate ·OH via Fenton-like reactions in the TME. Moreover, NIR irradiation markedly enhanced the ·OH signal intensity (Fig. 3I), further supporting the role of the photothermal effect in promoting ·OH generation.

### 2.6 Cellular uptake

**Fig. 4:**
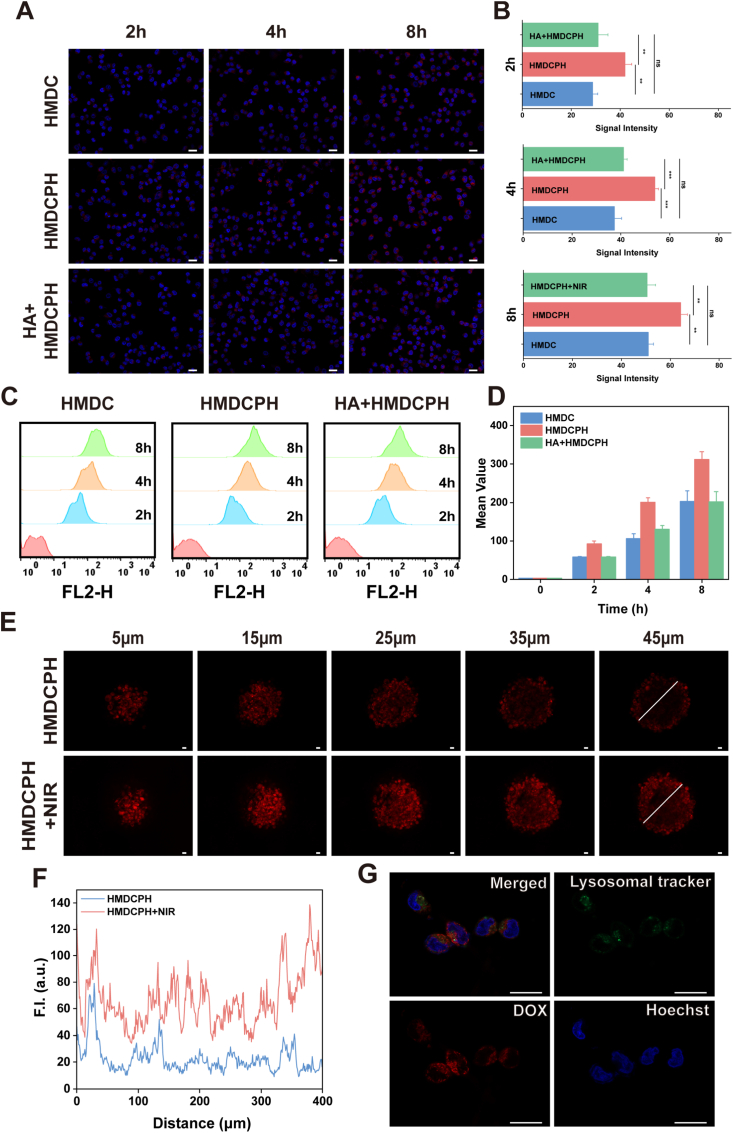
CLSM images (A) and fluorescence quantification (B) of cellular uptake in SW1990 cells treated with HMDC, HMDCPH, or HA + HMDCPH (scale bar: 20 μm). FCM uptake analysis (C) and mean analysis (D) of SW1990 cells treated with HMDC, HMDCPH, or HA + HMDCPH. CLSM images of fluorescence penetration (E) and fluorescence intensity analysis (F) in 3D tumor spheroids in vitro after treatment with HMDCPH or HMDCPH + NIR (scale bar: 20 μm). Colocalization of HMDCPH NPs with lysosomes (G) (scale bar: 20 μm). ∗*p* < 0.05, ∗∗*p* < 0.01, ∗∗∗*p* < 0.001, and ∗∗∗∗*p* < 0.0001.

The clinical application of nanomedicines depends on their selective recognition by tumor cells and efficient cellular internalization [33]. Confocal laser scanning microscopy (CLSM) showed that intracellular DOX fluorescence intensity gradually increased over time in all treatment groups. Compared with the HMDC group, HMDCPH-treated cells exhibited markedly stronger intracellular fluorescence (Fig. 4A and B). When cells were pretreated with free HA to block CD44 receptors, the fluorescence signal was significantly reduced. These results indicate that the efficient internalization of HMDCPH was mediated by HA-specific recognition. Flow cytometry (FCM) analysis further supported these findings (Fig. 4C and D). Three-dimensional (3D) SW1990 tumor spheroids were used to evaluate nanoparticle penetration. Z-stack scans showed that HMDCPH alone produced detectable red fluorescence within the spheroids, whereas NIR irradiation significantly increased both fluorescence intensity and maximum penetration depth (Fig. 4E and F). The enhanced penetration was likely associated with the photothermal effect of PDA. The photothermal effect may loosen the dense fibrotic extracellular matrix (ECM), thereby facilitating nanoparticle diffusion into deeper tumor regions [[34], [35], [36]]. In addition, CLSM imaging after 8 h of incubation showed clear colocalization between DOX and the lysosomal tracker (Fig. 4G). This colocalization indicates efficient cellular internalization, which may contribute to subsequent cytotoxic effects.

### 2.7 In vitro antitumor efficacy

**Fig. 5:**
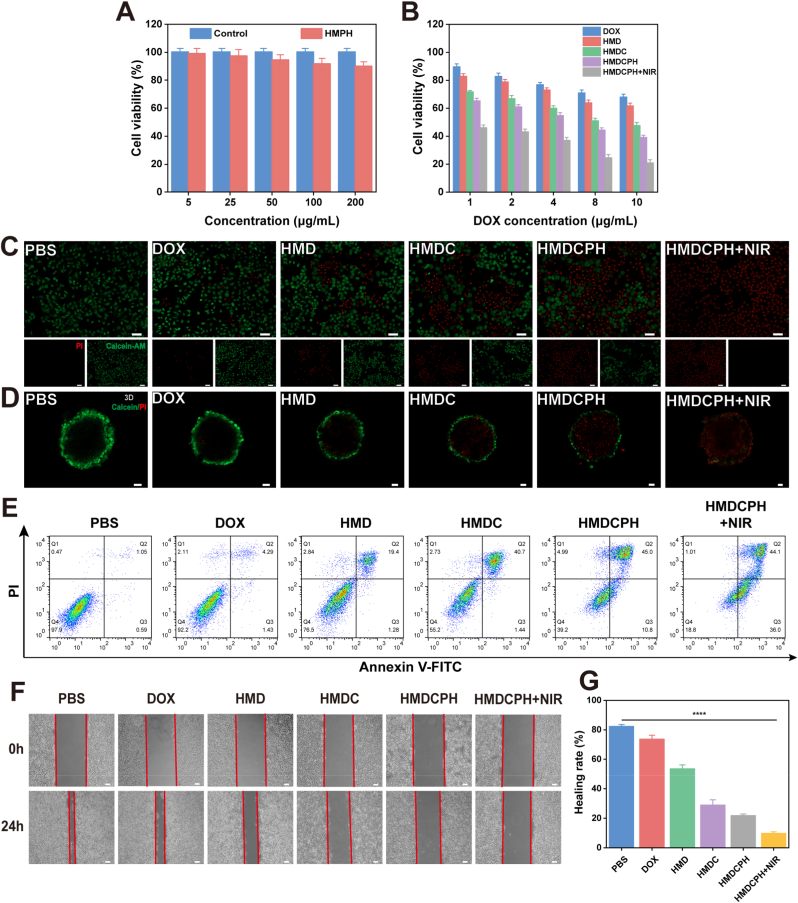
HPDE6C7 cell viability after treatment with different concentrations of HMPH NPs (A). Evaluation of therapeutic outcomes under various treatment regimens, including SW1990 cell viability (B), CLSM images of Calcein-AM/PI-stained SW1990 cells (C), CLSM images of Calcein-AM/PI-stained 3D tumor spheroids (D), FCM analysis of apoptosis (E), wound healing images (F), and wound healing rates (G) (scale bar: 50 μm). ∗*p* < 0.05, ∗∗*p* < 0.01, ∗∗∗*p* < 0.001, and ∗∗∗∗*p* < 0.0001.

The CCK-8 assay was performed to evaluate the in vitro cytotoxicity of the nanoplatform. Even after exposure to a high concentration of HMPH NPs (200 μg mL^−1^) for 24 h, the viability of normal HPDE6C7 cells remained above 90%, demonstrating the excellent biocompatibility of HMPH nanocarriers (Fig. 5A). At an equivalent DOX concentration, the free DOX group showed the highest SW1990 cell viability. In contrast, the HMDC and HMDCPH groups significantly reduced cell viability, whereas the HMDCPH + NIR group exhibited the lowest cell viability and the strongest cytotoxic effect against SW1990 cells (Fig. 5B).

To further validate the tumor cell death induced by the nanoplatform, live cells (green) and dead cells (red) were visualized using calcein-acetoxymethyl ester/propidium iodide (Calcein-AM/PI) staining. The PBS, DOX, and HMD groups exhibited limited cell death. In contrast, the HMDC and HMDCPH groups showed substantial cell death, whereas the HMDCPH + NIR group exhibited an almost complete loss of viable cells (Fig. 5C). Similar results were observed in 3D SW1990 tumor spheroids, in which HMDCPH + NIR induced extensive cell death throughout the spheroids (Fig. 5D). FCM analysis further confirmed that the HMDCPH + NIR group exhibited the highest apoptotic rate, with apoptosis rates of 1.64%, 5.74%, 20.68%, 42.14%, 55.8%, and 80.1% in the PBS, DOX, HMD, HMDC, HMDCPH, and HMDCPH + NIR groups, respectively (Fig. 5E). Migration and colony formation assays further demonstrated that HMDCPH + NIR most effectively inhibited tumor cell migration and colony formation (Fig. 5F, G and S7). Taken together, these results demonstrate that HMDCPH + NIR exhibited the strongest in vitro antitumor efficacy among all treatment groups.

### 2.8 Intracellular H_2_O_2_ production and calcium overload

**Fig. 6:**
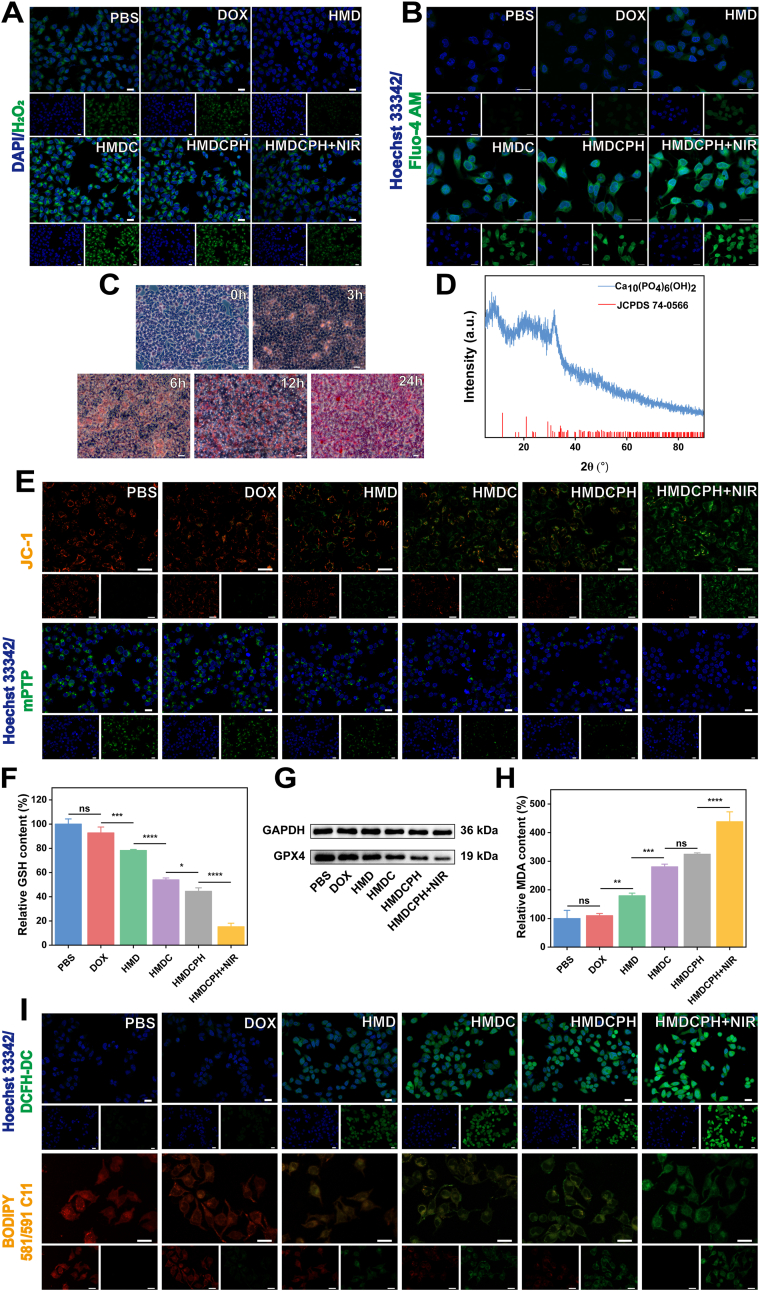
CLSM images of intracellular H_2_O_2_ (A) and Ca^2+^ (B) levels in SW1990 cells under various therapeutic regimens (scale bar: 20 μm). Identification of exocytosed products from SW1990 cells incubated with HMDCPH for different durations, with Alizarin Red staining showing calcium-rich regions in red (C) (scale bar: 100 μm). XRD patterns of cellular secretions collected from HMDCPH-treated cells (D). Under different therapeutic regimens, CLSM images of MMP and mPTP opening (E) (scale bar: 20 μm), intracellular GSH levels (F), GPX4 protein expression by Western blotting (G), MDA levels (H), and CLSM images of intracellular ROS and LPO levels (I) were evaluated (scale bar: 20 μm). ∗*p* < 0.05, ∗∗*p* < 0.01, ∗∗∗*p* < 0.001, and ∗∗∗∗*p* < 0.0001. (For interpretation of the references to color in this figure legend, the reader is referred to the Web version of this article.)

Intracellular H_2_O_2_ levels across treatment groups were detected with the BBoxiProbe® O16 green fluorescent probe. As shown in Fig. 6A, cells treated with CaO_2_-loaded nanoparticles, including HMDC and HMDCPH, exhibited significantly stronger green fluorescence than those in the other groups, indicating that CaO_2_-containing nanoparticles effectively elevated intracellular H_2_O_2_ levels. This increase was mainly attributed to abundant H_2_O_2_ generation from CaO_2_ within HMDCPH NPs under acidic TME conditions, accompanied by Ca^2+^ release. This self-supplied H_2_O_2_ provided sufficient substrate for subsequent redox reactions. The green fluorescence in the HMDCPH + NIR group was slightly attenuated, likely because the NIR-mediated photothermal effect accelerated H_2_O_2_ consumption. Meanwhile, intracellular Ca^2+^ levels across treatment groups were measured with the Fluo-4 AM green fluorescent probe. As shown in Fig. 6B, markedly stronger green fluorescence was detected in the HMDC, HMDCPH, and HMDCPH + NIR groups than in the control group, particularly in the HMDCPH + NIR group. These results demonstrate that HMDCPH NPs induced intracellular calcium overload, which was further intensified by the NIR-mediated photothermal effect. Moreover, Alizarin Red staining showed that calcified nodule formation, indicated by red-stained regions, progressively increased in SW1990 cells with prolonged treatment time (Fig. 6C). The XRD patterns of cellular secretions collected after HMDCPH treatment matched the standard pattern of hydroxyapatite [Ca_10_(PO_4_)_6_(OH)_2_, JCPDS No. 74-0566] (Fig. 6D), confirming the presence of calcified deposits. The HMDCPH-induced Ca^2+^ overload was further supported by Alizarin Red staining and by XRD-confirmed hydroxyapatite formation.

### 2.9 Cascade-amplified ferroptosis-related oxidative damage

Accumulating evidence indicates that intracellular Ca^2+^ overload can exceed mitochondrial buffering capacity, thereby causing irreversible mitochondrial damage and subsequent dysfunction [37,38]. In addition, mitochondrial function is closely associated with cellular redox homeostasis [39,40]. The effects of HMDCPH NPs on mitochondrial function in SW1990 cells were evaluated by monitoring mitochondrial membrane potential (MMP) and mitochondrial permeability transition pore (mPTP) opening. MMP was assessed using the JC-1 probe, in which a transition from red to green fluorescence indicates MMP depolarization. MMP progressively decreased across the treatment groups in the following order: PBS, DOX, HMD, HMDC, HMDCPH, and HMDCPH + NIR. Notably, the most pronounced MMP reduction was observed in the HMDCPH + NIR group (Fig. 6E). Concurrently, the green fluorescence signal in the mPTP assay progressively decreased, indicating increased mPTP opening. This change in mPTP opening was consistent with MMP depolarization. Notably, the highest intracellular Ca^2+^ level was observed in the HMDCPH + NIR group (Fig. 6B), coinciding with the greatest MMP depolarization and the highest degree of mPTP opening. These findings demonstrate that HMDCPH NPs effectively induced mitochondrial dysfunction, which was further aggravated by NIR irradiation.

Disruption of redox homeostasis in cancer cells, accompanied by enhanced LPO, is a key prerequisite for ferroptosis [41]. GSH, a major intracellular antioxidant, was quantified under various therapeutic regimens. As shown in Fig. 6F, intracellular GSH levels progressively decreased in the following order: PBS, DOX, HMD, HMDC, HMDCPH, and HMDCPH + NIR. GSH serves as an essential cofactor for GPX4, and its depletion can compromise GPX4 activity, thereby intensifying LPO and facilitating ferroptosis [42]. Moreover, GPX4 downregulation is widely recognized as a hallmark of ferroptosis [43]. Western blot analysis revealed the lowest GPX4 expression in the HMDCPH + NIR group, followed by the HMDCPH group (Fig. 6G and S8). Consistently, the HMDCPH + NIR group exhibited the highest level of malondialdehyde (MDA), a representative end product of lipid peroxidation, indicating the most severe lipid peroxidative damage (Fig. 6H). To further evaluate the oxidative stress induced by HMDCPH NPs, intracellular ROS and LPO levels were measured using 2′,7′-dichlorodihydrofluorescein diacetate (DCFH-DA) and BODIPY 581/591 C11, respectively. ROS oxidized DCFH-DA to generate the green fluorescent product 2′,7′-dichlorofluorescein (DCF), whereas increased LPO was reflected by a shift in BODIPY fluorescence emission from 590 nm (red) to 510 nm (green). As shown in Fig. 6I, DCF green fluorescence progressively increased from the PBS group to the HMDCPH + NIR group, whereas BODIPY staining showed a decrease in red fluorescence and a corresponding increase in green fluorescence. Among all groups, the HMDCPH + NIR group displayed the strongest ROS and LPO signals, indicating the most severe oxidative damage. These changes were consistent with the elevated intracellular H_2_O_2_ and Ca^2+^ levels and aggravated mitochondrial dysfunction described above (Fig. 6A, B and E). Taken together, these findings demonstrate that CaO_2_-loaded HMDCPH NPs, especially under NIR irradiation, markedly promoted GSH depletion, suppressed GPX4 expression, and enhanced ROS generation and lipid peroxidation, thereby exacerbating ferroptosis-related oxidative damage.

### 2.10 In vivo imaging

**Fig. 7:**
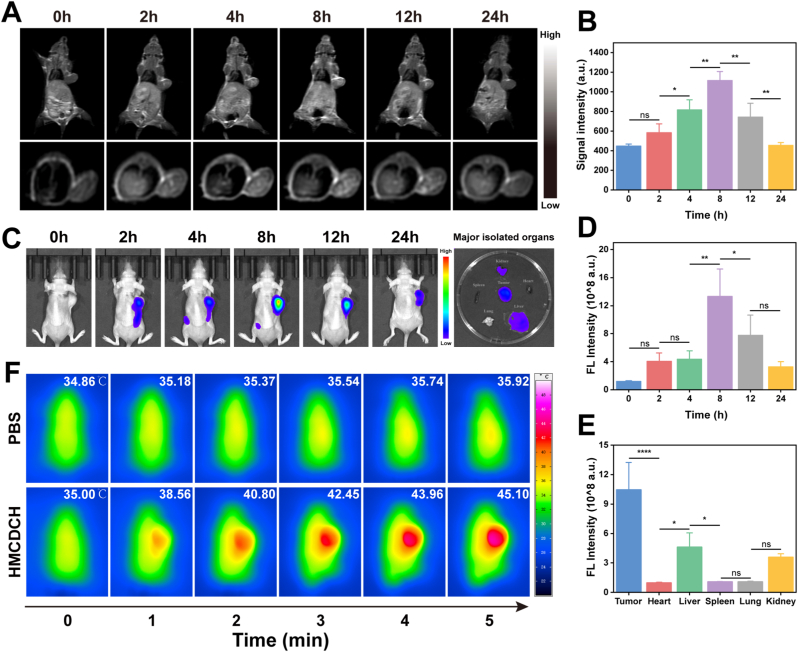
T_1_-weighted MR images of nude mice acquired within 24 h after HMDCPH injection (A) and the corresponding quantitative analysis of MR signal intensity (B). In vivo fluorescence images obtained within 24 h after HMDCPH injection, together with ex vivo fluorescence images of excised tissues (C). Quantitative analysis of fluorescence signals in tumors of living mice (D) and excised tissues (E). Thermal mapping of PBS- and HMDCPH-injected mice under NIR irradiation (F). *∗p* < 0.05, *∗∗p* < 0.01, *∗∗∗p* < 0.001, and *∗∗∗∗p* < 0.0001.

The in vivo MR imaging capability of HMDCPH NPs was evaluated in tumor-bearing nude mice. Fig. 7A and B show enhanced MR contrast at the tumor site 2 h after HMDCPH injection, indicating rapid accumulation of HMDCPH NPs at the tumor site and subsequent responsive degradation. Notably, the MR signal peaked at 8 h and then gradually declined. These findings demonstrate the promising potential of HMDCPH NPs for T_1_-weighted MR imaging.

In vivo fluorescence imaging was performed to assess the tumor accumulation of HMDCPH NPs. Fig. 7C–E shows a time-dependent increase in tumor fluorescence after injection, with the strongest signal observed at 8 h post-injection, followed by a gradual decrease, a trend consistent with the MRI results described above. Ex vivo imaging at 24 h post-injection revealed higher fluorescence intensity in excised tumors than in major organs. The contents of Mn and Ca in harvested tumors and major organs were further determined by inductively coupled plasma mass spectrometry (ICP-MS). The results revealed predominant Mn and Ca accumulation in tumor tissues, liver, and kidneys, in agreement with the ex vivo fluorescence imaging results (Fig. S9). These findings further support the tumor-targeted accumulation and in vivo biodistribution profile of HMDCPH NPs and suggest that their degradation products may undergo hepatic and renal clearance.

### 2.11 In vivo photothermal performance

To evaluate the in vivo photothermal performance of HMDCPH NPs, tumor-bearing nude mice received PBS or HMDCPH NPs via tail vein injection, followed by real-time temperature monitoring during NIR irradiation. The temperature at the tumor site in the PBS-treated group reached only 35.92 °C, indicating that NIR irradiation alone did not cause tissue overheating. By comparison, the tumor temperature in the HMDCPH + NIR group rapidly rose to 45.10 °C, surpassing the therapeutic threshold of 42 °C for photothermal therapy (Fig. 7F). These findings demonstrate that HMDCPH NPs exhibit strong in vivo photothermal conversion capability.

### 2.12 In vivo antitumor efficacy

**Fig. 8:**
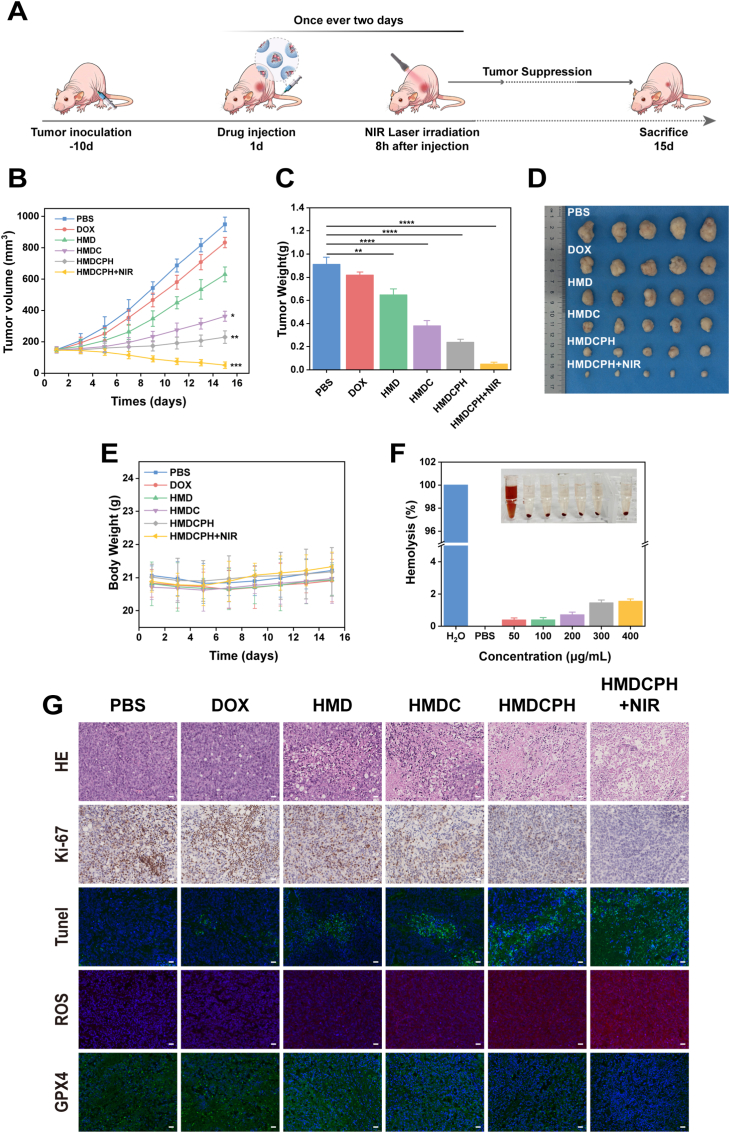
In vivo antitumor treatment design (A). Tumor volume (B), tumor weight (C), photographs of excised tumors (D), and body weight variation (E) in nude mice subjected to various treatments. Hemolytic activity of HMDCPH NPs against human red blood cells (F). Representative histological and fluorescence analyses of tumors across treatment groups (G) (scale bar: 20 μm). *∗p* < 0.05, *∗∗p* < 0.01, *∗∗∗p* < 0.001, and *∗∗∗∗p* < 0.0001. (For interpretation of the references to color in this figure legend, the reader is referred to the Web version of this article.)

Encouraged by the prominent in vitro antitumor activity of HMDCPH NPs, their in vivo antitumor efficacy was further evaluated in female BALB/c nude mice (Fig. 8A). After 15 days of treatment, the average tumor volume and tumor weight in the HMDCPH + NIR group were approximately 51.42 mm^3^ and 0.04 g, respectively, which were markedly lower than those in the other treatment groups (Fig. 8B–D). The tumor growth curves further confirmed that tumor growth was effectively inhibited in the HMDCPH + NIR group throughout the treatment period (Fig. 8B). Moreover, no treatment-associated deaths were observed in any group, and mouse body weights remained relatively stable (Fig. 8E), suggesting good tolerability of the therapeutic strategy.

To further investigate the in vivo therapeutic efficacy of HMDCPH NPs, tumor tissues were subjected to histological and immunofluorescence analyses. H&E and TUNEL staining revealed extensive tumor cell death in the HMDCPH + NIR group, whereas Ki-67 staining showed reduced tumor cell proliferation, indicating effective tumor growth inhibition (Fig. 8G). In addition, immunofluorescence analysis showed that this group exhibited the highest ROS level and the lowest GPX4 expression among all groups (Fig. 8G), which was consistent with the in vitro findings. These findings suggest that HMDCPH NPs combined with NIR irradiation effectively induced ferroptosis-related oxidative damage in vivo and produced a synergistic antitumor effect through integrated chemotherapy and photothermal therapy.

### 2.13 Biosafety of HMDCPH NPs

Biosafety is a key consideration for the biomedical applications of nanomaterials. Here, the hemocompatibility of HMDCPH NPs was evaluated using a hemolysis assay. As shown in Fig. 8F, all tested concentrations resulted in hemolysis rates below 5%, indicating favorable blood compatibility. Echocardiographic analysis showed a reduced left ventricular ejection fraction (EF) in the DOX group, whereas EF remained within the normal range in tumor-bearing mice treated with PBS or HMDCPH (Fig. S10A and B). This result suggests that HMDCPH NPs may mitigate DOX-associated cardiotoxicity and are well tolerated in vivo. Blood biochemical tests further showed that HMDCPH treatment did not cause apparent changes in hepatic markers (ALT and AST) or renal markers (UREA and CREA) compared with PBS treatment (Fig. S11), suggesting no apparent hepatic or renal toxicity. Consistently, H&E staining of major organs showed no apparent pathological injury or inflammatory infiltration across all groups (Fig. S12). These results support the favorable biosafety of HMDCPH NPs.

## 3 Conclusion

In conclusion, we developed a tumor-targeted multifunctional nanoplatform, HMDCPH, capable of efficiently co-delivering DOX and CaO_2_. In the acidic and GSH-rich TME, HMDCPH underwent stimuli-responsive degradation. The released Mn^2+^ not only served as a T_1_-weighted MRI contrast agent but also catalyzed ·OH generation via Fenton-like reactions. Meanwhile, CaO_2_ decomposition supplied H_2_O_2_ and released Ca^2+^, and the exogenous Ca^2+^ induced mitochondrial Ca^2+^ overload and dysfunction, which synergized with H_2_O_2_ supply to further elevate intracellular ROS levels. The combination of GSH depletion and ROS accumulation aggravated cellular oxidative stress, whereas the photothermal effect under NIR irradiation further amplified this damage. These combined effects ultimately enhanced lipid peroxidation and intensified ferroptosis-related oxidative damage, as further supported by the downregulation of GPX4, a key ferroptosis-suppressing enzyme. Both in vitro and in vivo results demonstrated that HMDCPH + NIR exerted potent synergistic antitumor activity. Overall, this study presents a nanoplatform-based synergistic therapeutic strategy that integrates chemotherapy, photothermal therapy, and ferroptosis-related oxidative damage amplification to overcome chemoresistance in pancreatic cancer.

## 4 Materials and methods

### 4.1 Materials

Ammonia solution (NH_3_·H_2_O, 28%), tetraethyl orthosilicate (TEOS), cetyltrimethylammonium bromide (CTAB), potassium permanganate (KMnO_4_), sodium carbonate (Na_2_CO_3_), doxorubicin (DOX), anhydrous calcium chloride (CaCl_2_), polyvinylpyrrolidone (PVP), hyaluronic acid (HA), 1-(3-dimethylaminopropyl)-3-ethylcarbodiimide hydrochloride (EDC), N-hydroxysuccinimide (NHS), dopamine hydrochloride (DA), DTNB, reduced glutathione (GSH), Ti(SO_4_)_2_, and methylene blue (MB) were procured from Aladdin Chemistry Co., Ltd. 30% H_2_O_2_ and the lysosomal tracker were supplied by Sinopharm Chemical Reagent Co., Ltd. and Dalian Meilun Biotechnology Co., Ltd., respectively. The H_2_O_2_, ROS (DCFH-DA), GSH/GSSG, MDA, and mPTP assay kits, JC-1 and Fluo-4 calcium probes, LPO probe (BODIPY 581/591 C11), and CCK-8, Calcein-AM/PI cell viability, and Alizarin Red staining kits were provided by Beyotime Biotechnology Co., Ltd. GPX4 and GAPDH polyclonal antibodies were acquired from Selleckchem and Proteintech, respectively. SW1990 and HPDE6C7 cell lines were supplied by the Shanghai Cell Bank of the Chinese Academy of Sciences.

### 4.2 Preparation of HM-MnO_2_@DOX/CaO_2_@PDA/HA NPs

#### 4.2.1 Preparation of HM-MnO_2_ NPs

SiO_2_ templates were prepared by stirring anhydrous ethanol (71.4 mL), water (10 mL), NH_3_·H_2_O (1.625 mL), and TEOS (3 mL) at 30 °C for 2 h. SiO_2_ templates (100 mg) were dispersed in water (12 mL), followed by the addition of anhydrous ethanol (30 mL), water (38 mL), NH_3_·H_2_O (2.3 mL), and CTAB (300 mg). After stirring for 0.5 h, KMnO_4_ (300 mg) was added, and the mixture was further stirred for 6 h. The particles were redispersed in an aqueous Na_2_CO_3_ solution (20 mL, 424 mg) and reacted at 50 °C for 12 h, followed by refluxing in methanol (10 mL) and NH_3_·H_2_O (1 mL) at 70 °C for 48 h. Finally, the products were washed and lyophilized to obtain HM-MnO_2_ NPs.

#### 4.2.2 Preparation of HM-MnO_2_@DOX/CaO_2_ NPs

HM-MnO_2_ NPs (10 mg) and DOX (5 mg) were dispersed in ethanol (10 mL) and stirred overnight to obtain HM-MnO_2_@DOX NPs. CaCl_2_ (50 mg) dissolved in anhydrous ethanol (5 mL) was blended with the above dispersion (5 mL) and stirred for 6 h. PVP (85 mg) and NH_3_·H_2_O (0.5 mL) were added for further reaction over 1.5 h. H_2_O_2_ (0.15 mL) was subsequently introduced at a flow rate of 0.2 mL h^−1^. HM-MnO_2_@DOX/CaO_2_ NPs were collected after washing with ethanol and drying.

#### 4.2.3 Preparation of HM-MnO_2_@DOX/CaO_2_@PDA/HA NPs

EDC and NHS were added to the HA solution under stirring at a molar ratio of HA:EDC:NHS = 1:3:3. HM-MnO_2_@DOX/CaO_2_ NPs were dispersed in anhydrous ethanol (10 mL) containing NH_3_·H_2_O (0.15 mL) and DA (2 mg), and the dispersion was maintained under dark conditions with stirring for 2 h. Following dropwise addition of the activated HA solution (0.2 mL), the mixture was stirred overnight to obtain HM-MnO_2_@DOX/CaO_2_@PDA/HA NPs.

### 4.3 Characterization

Nanoparticle morphology, structure, and elemental distribution were characterized by TEM coupled with EDS (Thermo Fisher Talos F200X, USA). BET analysis was applied to evaluate the surface properties and pore texture (Micromeritics ASAP 2460, USA). Zeta potential and hydrodynamic size were measured using DLS (Zetasizer Nano ZS90, Malvern, UK). By tracking the hydrodynamic size in PBS and RPMI-1640 medium over the course of a week, the colloidal stability of HMDCPH NPs was assessed. Elemental valence states, crystal structure, and characteristic absorption peaks were analyzed by XPS (K-Alpha, Thermo Fisher Scientific, Japan), XRD (Bruker D8 Advance, Germany), and FT-IR (FT-IR 6800 JASCO, Marseille, France), respectively. UV–vis spectroscopy (UV-2600I, Shimadzu, Japan) was employed to verify drug loading.

### 4.4 Drug loading and in vitro release study

The DOX standard curve was established based on UV–vis absorbance at 480 nm. DOX was loaded into HM-MnO_2_ during the synthesis of HMDCPH. The supernatant obtained after repeated centrifugation was used for absorbance measurement, and the DL and EE values were then computed.

$$\text{DL}\left(\%\right)=\frac{\text{weight}\;\text{of}\;\text{the}\;\text{drug}\;\text{in}\;\text{NPs}}{\text{weight}\;\text{of}\;\text{NPs}}\times 100\%$$

$$\text{EE}\left(\%\right)=\frac{\text{weight}\;\text{of}\;\text{the}\;\text{drug}\;\text{in}\;\text{NPs}}{\text{weight}\;\text{of}\;\text{the}\;\text{drug}\;\text{in}\;\text{feed}}\times 100\%$$

To evaluate DOX release, HMDCPH NPs containing equivalent amounts of DOX were sealed in dialysis bags and incubated in PBS under different conditions (pH 7.4 or 5.5, 0 or 5 mM GSH, and 150 U·mL^−1^ hyaluronidase). The samples were shaken at 130 rpm in the dark. At predetermined intervals, 2 mL of the release medium was withdrawn for absorbance measurement at 485 nm and replaced with an equal volume of fresh medium.

### 4.5 Evaluation of in vitro MRI performance

HMDCPH NPs with various Mn concentrations (0.1−1.0 mM) were incubated in PBS under different conditions (pH 7.4 or 5.5, 0 or 5 mM GSH, and 150 U·mL^−1^ hyaluronidase). The r_1_ relaxivity of the supernatants was measured using a 0.5 T NM120 analyzer, and T_1_-weighted MR imaging was conducted on a 3.0 T MRI scanner.

### 4.6 Evaluation of in vitro photothermal properties

The photothermal temperature evolution of HMDCPH and HMDCH NP dispersions was tracked using a thermal imaging camera during 808 nm laser irradiation (1 W cm^−2^, 10 min). Subsequently, the photothermal behavior of HMDCPH NPs was further investigated under varying laser intensities (0.5−2.5 W cm^−2^) and nanoparticle concentrations (0−250 μg mL^−1^). Five repeated irradiation–cooling cycles were performed to examine the photothermal cycling stability. The photothermal conversion efficiency (η) was determined as follows:

$$\eta =\frac{\text{hA}\left({\Delta T}_{\max,\text{mix}}‐{\Delta T}_{\max,H_{2}O}\right)}{I\left(1‐10^{‐A_{808}}\right)}$$

### 4.7 In vitro detection of GSH depletion, H_2_O_2_ generation, and ·OH production

GSH depletion was determined via the DTNB method. CaO_2_, HM, and HMC (200 μg mL^−1^) were mixed with GSH (50 μM) for 2 h and then centrifuged. After the supernatants were mixed with DTNB solution, the absorbance was recorded at 412 nm. GSH + H_2_O_2_ and GSH alone were used as the positive and negative controls, respectively. In addition, HMCPH NPs at various concentrations (0–400 μg mL^−1^) were mixed with GSH, and residual GSH was quantified by the same DTNB method.

H_2_O_2_ generation was assessed using Ti(SO_4_)_2_ and a commercial H_2_O_2_ assay kit. CaO_2_, HM, and HMC were dispersed in slightly acidic solutions, and the supernatants were mixed with Ti(SO_4_)_2_ reagent (1 mg mL^−1^) before absorbance measurement at 410 nm. Ti(SO_4_)_2_ + H_2_O_2_ and Ti(SO_4_)_2_ alone were used as the positive and negative controls, respectively. For further investigation, HMC and HMCPH were incubated under different pH conditions, and H_2_O_2_ generation was analyzed using a commercial H_2_O_2_ assay kit.

·OH generation was evaluated using the MB bleaching assay and ESR spectroscopy. HMPH or HMCPH dispersions (50 μg mL^−1^) were incubated in a mixed NaCl/NaHCO_3_ solution, and the resulting supernatants were collected and subsequently mixed with MB solution (20 μM) in the presence or absence of H_2_O_2_ and/or NIR irradiation. The absorbance at 665 nm was then measured. For ESR analysis, HMCPH NPs were dispersed in solutions with different pH values and GSH concentrations, incubated for 30 min, irradiated with an NIR laser or left untreated, and then analyzed by ESR spectroscopy.

### 4.8 Cellular uptake evaluation and lysosomal colocalization study

SW1990 cells were seeded in dishes and allowed to adhere overnight, followed by exposure to HMDC, HMDCPH, or HA + HMDCPH for 2, 4, or 8 h. At predefined time points, CLSM imaging and FCM analysis were performed. For lysosomal colocalization analysis, SW1990 cells were incubated with HMDCPH for 8 h and then labeled with Hoechst 33258 and LysoTracker before CLSM imaging. In addition, 3 × 10^3^ SW1990 cells were plated into ultra-low-attachment plates and cultured to form spheroids. HMDCPH was applied to the spheroids for 8 h with or without NIR exposure. The spheroids were transferred to dishes for CLSM imaging following treatment. Z-stack scans were recorded from the spheroid surface toward the center at 10 μm intervals and analyzed using ImageJ to evaluate nanoparticle penetration.

### 4.9 In vitro antitumor study

HPDE6C7 and SW1990 cells were seeded in 96-well plates and allowed to adhere overnight. HPDE6C7 cells were exposed to HMPH NPs at different concentrations for 24 h, whereas SW1990 cells were treated with DOX, HMD, HMDC, HMDCPH, or HMDCPH + NIR at different DOX-equivalent concentrations for 24 h. For the HMDCPH + NIR group, cells were treated with HMDCPH for 8 h and then irradiated with an 808 nm NIR laser (1 W cm^−2^, 10 min). After incubation with CCK-8 reagent, the absorbance at 450 nm was recorded.

$$\text{Cell}\;\text{viability}\left(\%\right)=\frac{{\text{OD}}_{\text{sample}}‐{\text{OD}}_{\text{blank}}}{{\text{OD}}_{\text{control}}‐{\text{OD}}_{\text{blank}}}\times 100\%$$

SW1990 cells were seeded in dishes and allowed to adhere overnight, and then subjected to various therapeutic regimens over a 24 h period. After treatment, Calcein-AM/PI labeling was performed prior to CLSM imaging. Cellular apoptosis was quantified by flow cytometry following Annexin V-FITC/PI dual staining. After formation, 3D cell spheroids were subjected to various therapeutic regimens prior to CLSM observation.

### 4.10 Cell migration and colony formation assays

SW1990 cells were seeded in 6-well plates and allowed to adhere overnight prior to scratching. Following this, various treatments were applied for 24 h, and wound healing was observed under a microscope. Colony formation was assessed by plating SW1990 cells in 6-well plates at 5 × 10^2^ cells/well, followed by 7 days of different interventions. The formed colonies were fixed, stained, and counted.

### 4.11 Detection of intracellular H_2_O_2_ production and Ca^2+^ levels

SW1990 cells were seeded in dishes overnight and then subjected to various therapeutic regimens for 12 h. Intracellular H_2_O_2_ and Ca^2+^ levels were detected using BBoxiProbe® O16 and Fluo-4 AM staining, respectively, followed by CLSM imaging. For calcium deposition assessment, cells were incubated with HMDCPH for 0–24 h, fixed with 95% ethanol, and stained with Alizarin Red before light microscopy observation. Specimens incubated with HMDCPH for 24 h were collected, rinsed, dehydrated and processed for XRD detection.

### 4.12 Mitochondrial dysfunction and ferroptosis-related analysis

SW1990 cells were seeded in dishes or 6-well plates overnight and then subjected to various therapeutic regimens for 12 h. MMP and mPTP opening were assessed via JC-1 and Calcein AM/CoCl_2_ staining with subsequent CLSM imaging. After cell collection, intracellular GSH/GSSG and MDA were quantified with commercial kits. Total protein from cell lysates was subjected to Western blotting for GPX4 detection. Intracellular ROS and LPO were labeled with DCFH-DA/Hoechst 33342 and BODIPY 581/591 C11, followed by CLSM observation.

### 4.13 In vivo imaging

Xenograft models were established by subcutaneous injection of 5 × 10^6^ SW1990 cells into the axilla of female BALB/c nude mice. When tumors reached the desired volume, mice received HMDCPH dispersion (100 μL) via tail vein injection. T_1_-weighted MR images were acquired using a 3.0 T MRI system at assigned time points (0, 2, 4, 8, 12, 24 h) to track tumor T_1_ signal changes. In addition, ICG-labeled nanoparticles were intravenously administered to mice, and fluorescent signal profiling was implemented at predetermined post-injection time points with a Caliper IVIS Spectrum imaging platform. Tumors and major organs were excised 24 h after administration and subjected to ex vivo fluorescence analysis and ICP-MS determination of Mn and Ca levels.

### 4.14 In vivo photothermal evaluation

After intravenous administration of HMDCPH NPs, the in vivo photothermal response was evaluated. Eight hours after injection, NIR irradiation (1 W cm^−2^, 5 min) was applied to the tumors, and thermal signals were captured using a FLIR T530 infrared imaging system.

### 4.15 In vivo antitumor study

Upon tumor progression to ∼150 mm^3^, mice were allocated to six cohorts (n = 5): PBS, DOX, HMD, HMDC, HMDCPH and HMDCPH + NIR. All regimens were delivered via the tail vein every other day, and tumors in the NIR group received 808 nm laser irradiation 8 h post-administration. During the treatment period, tumor growth and body weight were recorded. On day 15, tumors were excised and weighed. The tumor tissues were subsequently processed for H&E, TUNEL, and Ki-67 staining, as well as immunofluorescence analysis of ROS and GPX4.

### 4.16 Biosafety assessment

The hemocompatibility of HMDCPH NPs was tested using a hemolysis assay. HMDCPH NPs (50−400 μg mL^−1^) were added to red blood cells after they had been washed and resuspended in a 4% (v/v) suspension. The positive and negative controls were deionized water and PBS, respectively. Following incubation at 37 °C for 4 h, the supernatant was analyzed by measuring absorbance at 576 nm. The biosafety of HMDCPH NPs was further validated via cardiac ultrasound, blood biochemistry (ALT, AST, UREA, CREA), and histological examination of major organs.

### 4.17 Statistical analysis

Statistical evaluations were performed using GraphPad Prism 10.1.2 and Origin 2021, with statistical significance defined as *p* < 0.05.

## CRediT authorship contribution statement

**Pan Yang:** Conceptualization, Data curation, Writing – original draft. **Shuai He:** Methodology, Supervision. **Mingdong Xu:** Project administration. **Yanying Sun:** Visualization. **Jingyi Gao:** Investigation. **Jian Chen:** Formal analysis, Software. **Tongtong Niu:** Software. **Liguo Hao:** Funding acquisition, Resources, Writing – review & editing.

## Declaration of competing interest

The authors declare that they have no known competing financial interests or personal relationships that could have appeared to influence the work reported in this paper.

## Data Availability

Data will be made available on request.
