# Supplementary material for: MRI-enabled ferroptosis self-amplifying nanoplatform synergizes with photothermal therapy to enhance chemotherapeutic efficacy against pancreatic cancer
Source: Mater Today Bio. 2026 Jul 16;39:103469. doi: 10.1016/j.mtbio.2026.103469 (PMC13393574; doi:10.1016/j.mtbio.2026.103469)
Supplement: Multimedia component 1 [file mmc1.docx]

**MRI-Enabled Ferroptosis Self-Amplifying Nanoplatform Synergizes with Photothermal Therapy to Enhance Chemotherapeutic Efficacy against Pancreatic Cancer**

Pan Yang^a^, Shuai He^a^, Mingdong Xu^a^, Yanying Sun^a^, Jingyi Gao^a^, Jian Chen^a^, Tongtong Niu^a^, Liguo Hao^a, *^

a. Department of Molecular Imaging, School of Medical Technology, Qiqihar Medical University, Qiqihar, Heilongjiang 161006, PR China

* Corresponding author. E-mail address: haoliguo@qmu.edu.cn (L. Hao)


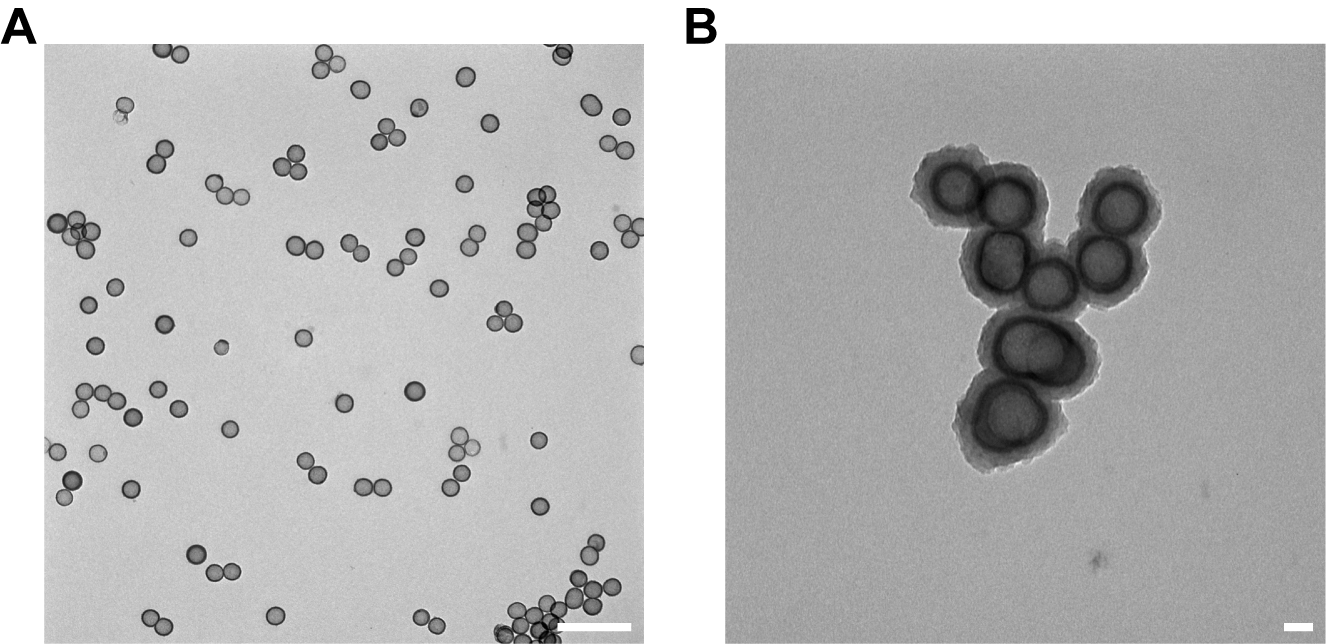


Fig. S1. TEM images of HMDC (A) (scale bar: 500 nm) and HMDCPH (B) (scale bar: 50 nm).


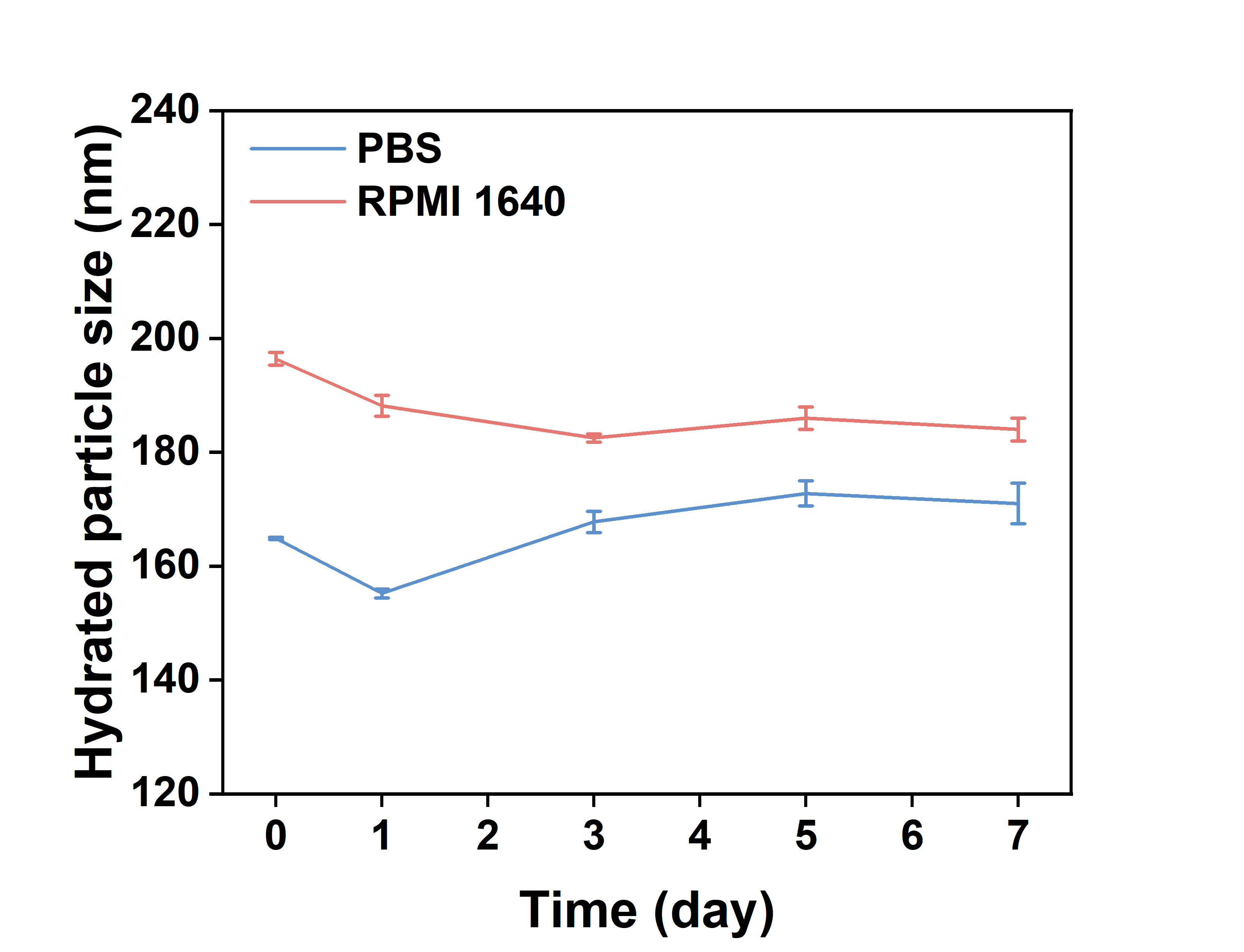


Fig. S2. Changes in the hydrodynamic diameter of HMDCPH NPs after incubation in PBS and RPMI 1640 medium for different durations.


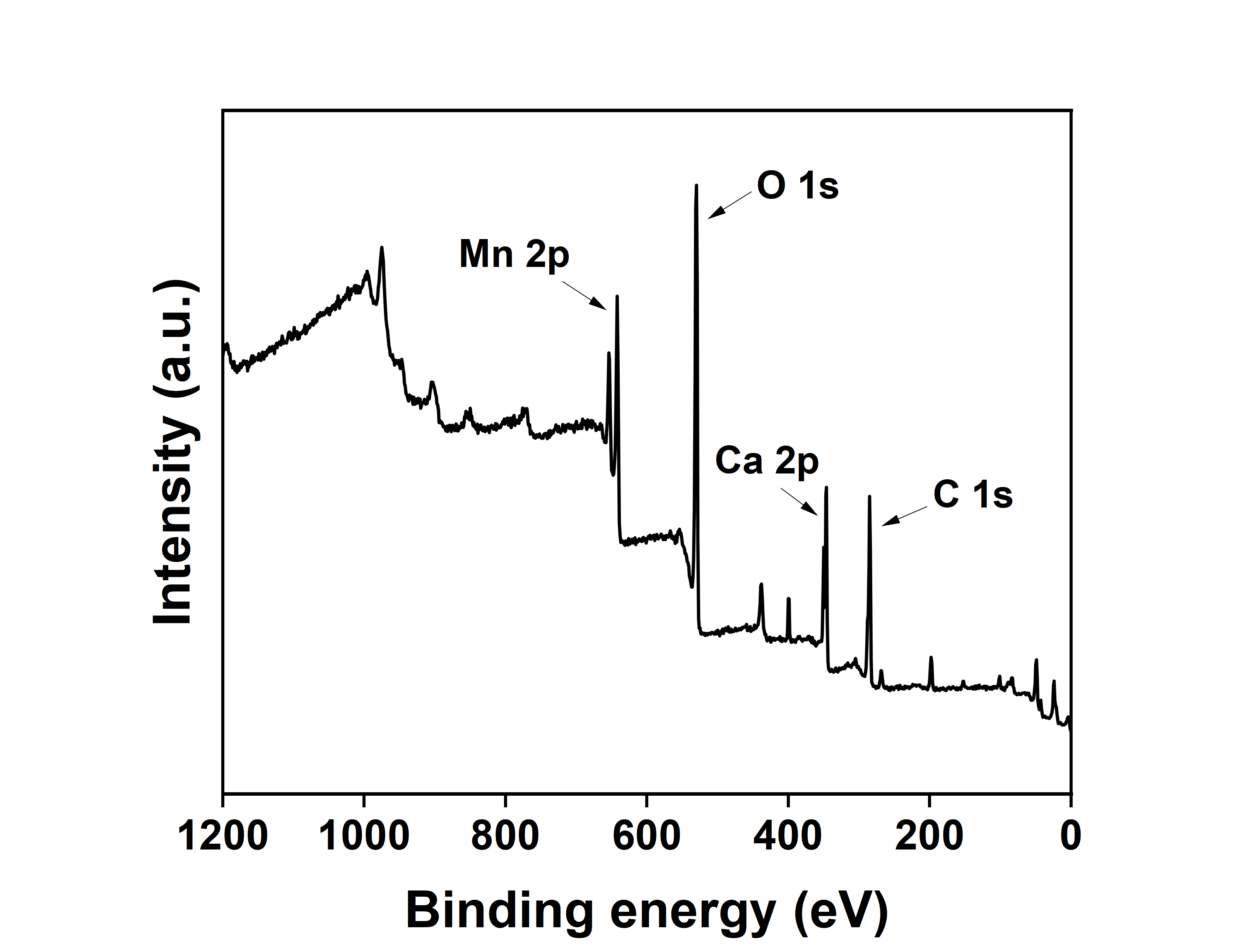


Fig. S3. XPS survey spectrum of HMDCPH.


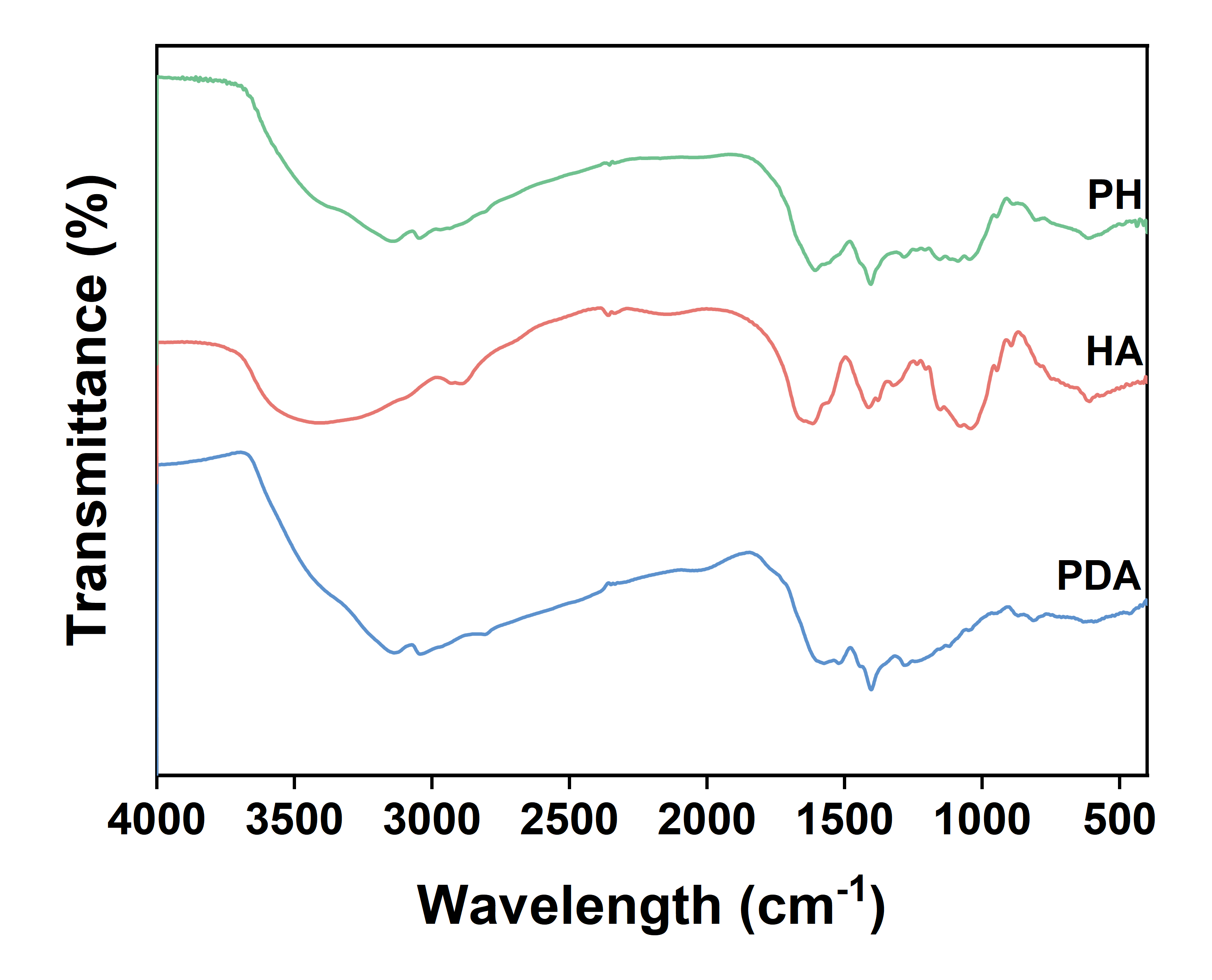


Fig. S4. FT-IR spectra of PDA, HA, and PH (PDA/HA).


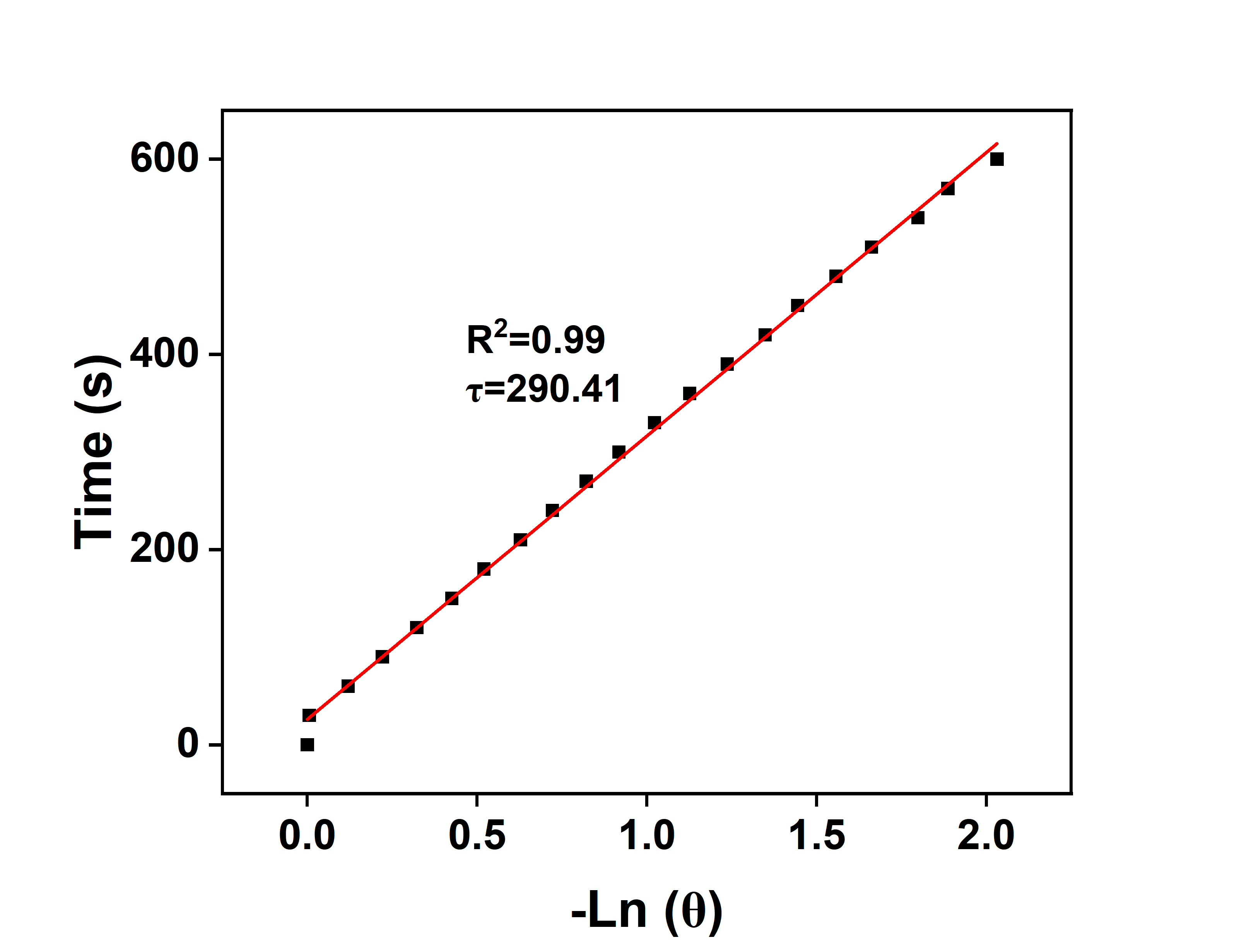


Fig. S5. Photothermal conversion efficiency fitting curve of HMDCPH NPs.


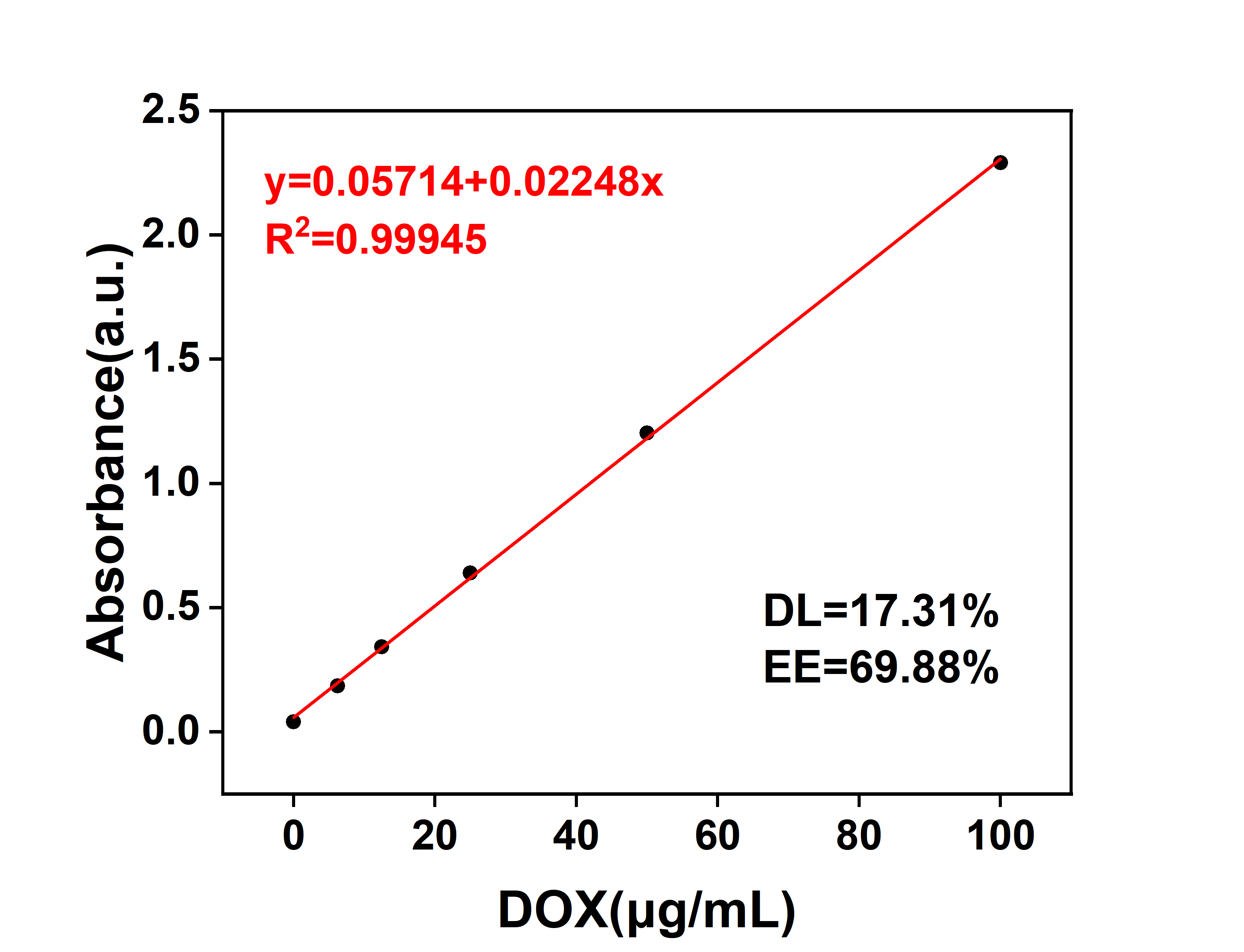


Fig. S6. UV-vis calibration curve of DOX and quantitative analysis of the drug loading (DL) and encapsulation efficiency (EE) of HMDCPH.


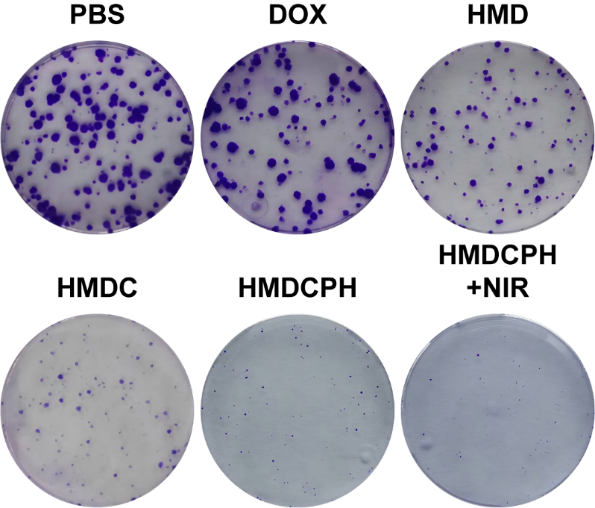


Fig. S7. Colony formation ability of cells under various therapeutic regimens.


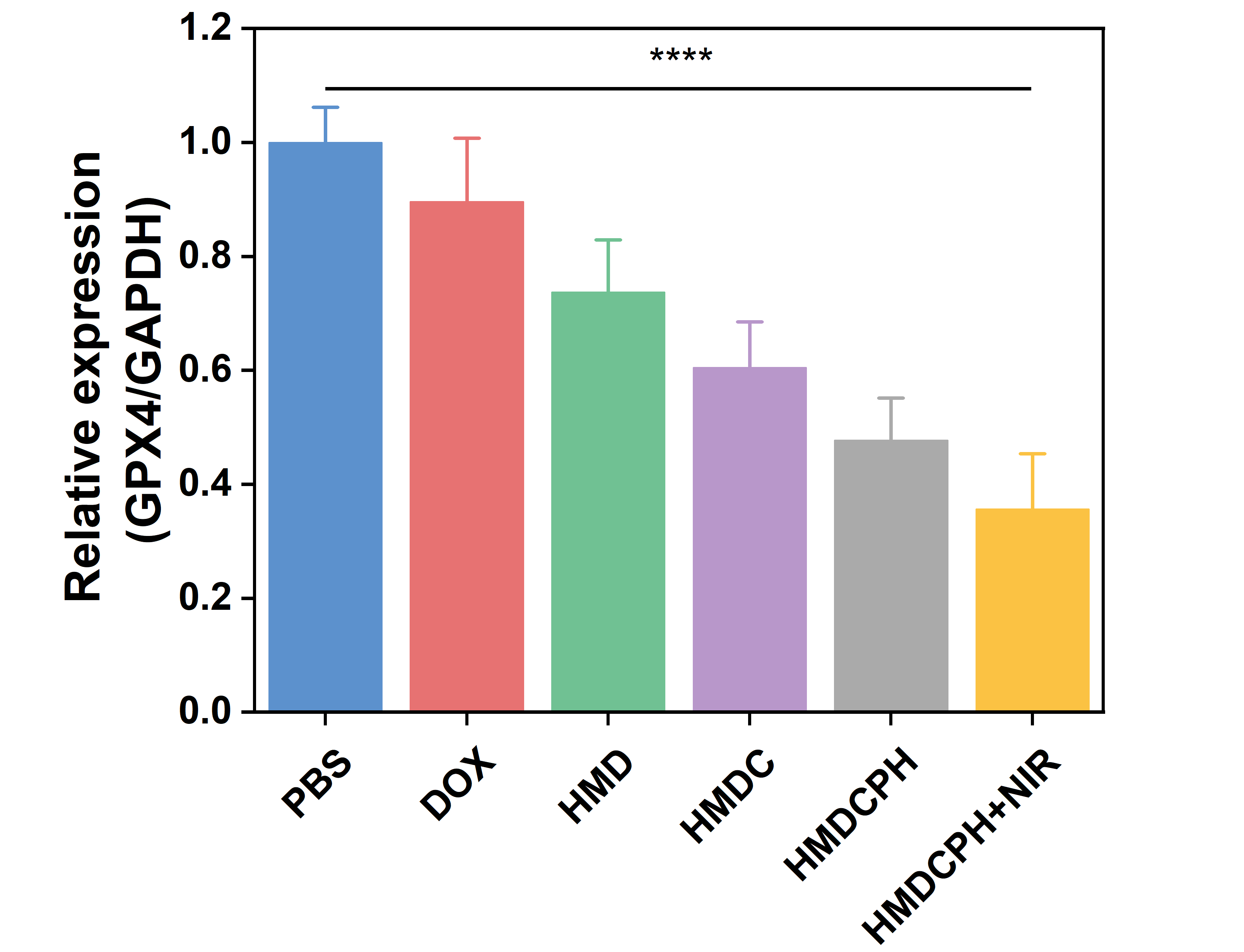


Fig. S8. Quantitative analysis of relative GPX4 expression normalized to GAPDH.


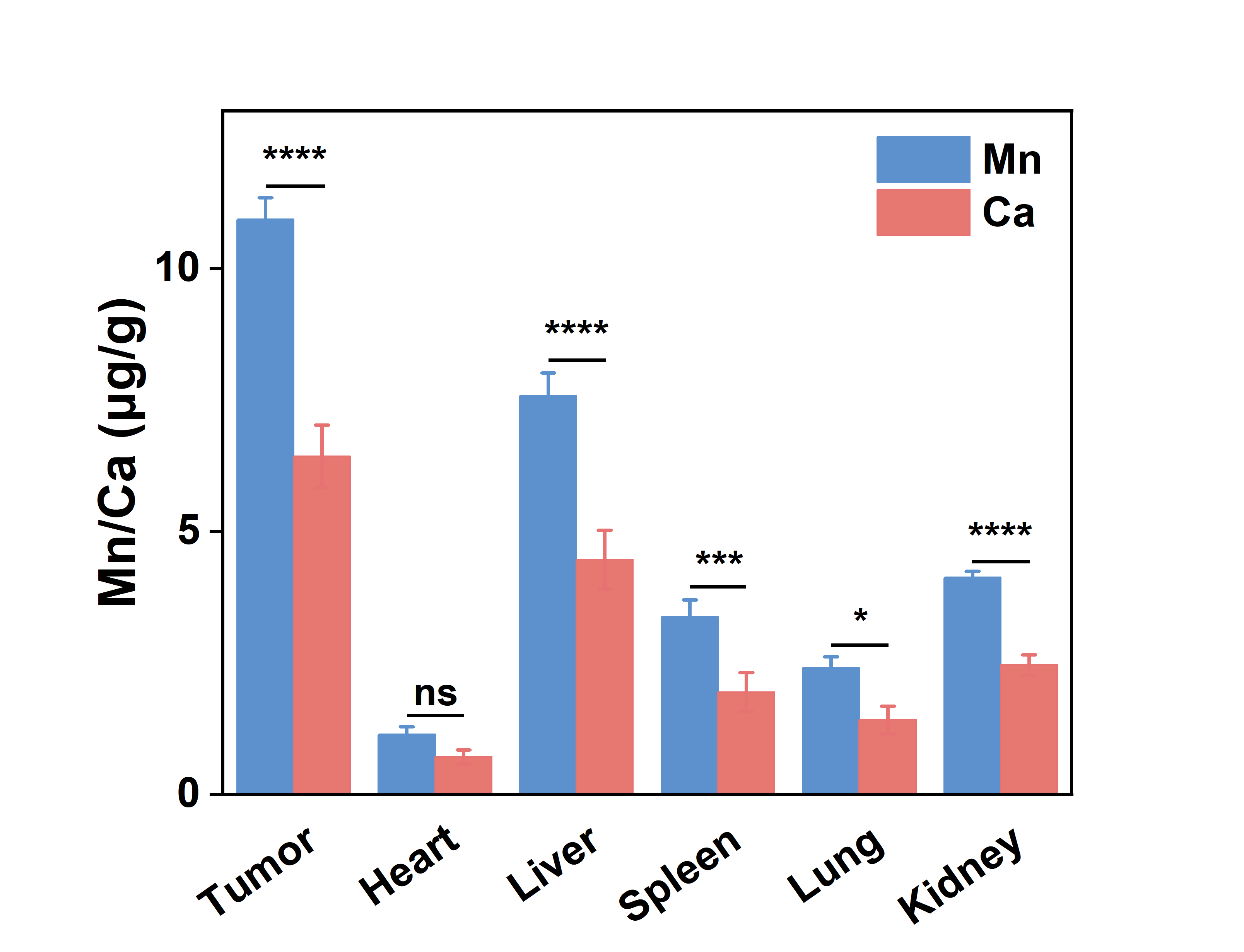


Fig. S9. Biodistribution of Mn and Ca in tumors and major organs 24 h after tail vein injection of HMDCPH.


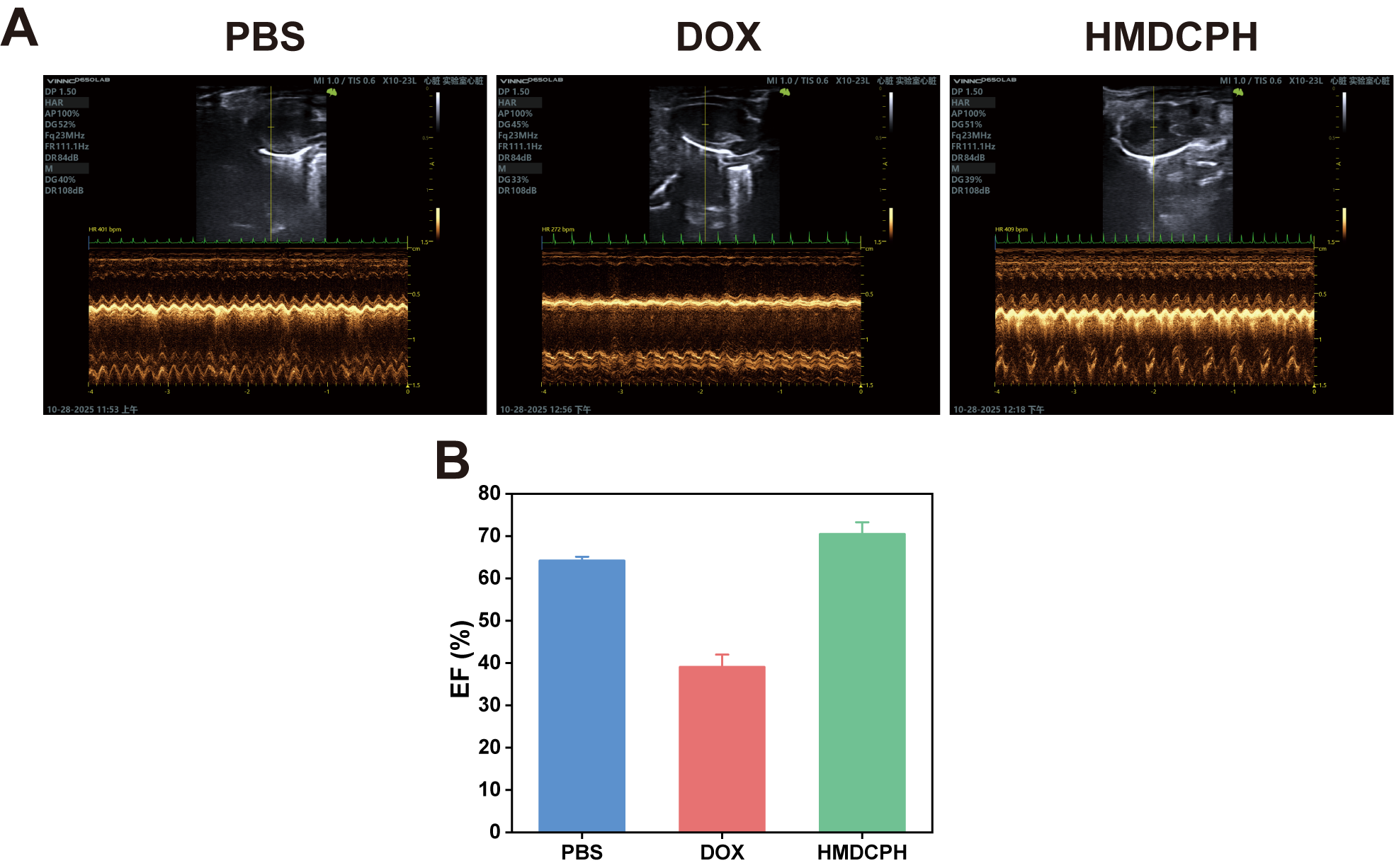


Fig. S10. Echocardiographic images (A) and quantitative analysis of left ventricular ejection fraction (EF) (B) in tumor-bearing nude mice from different treatment groups.


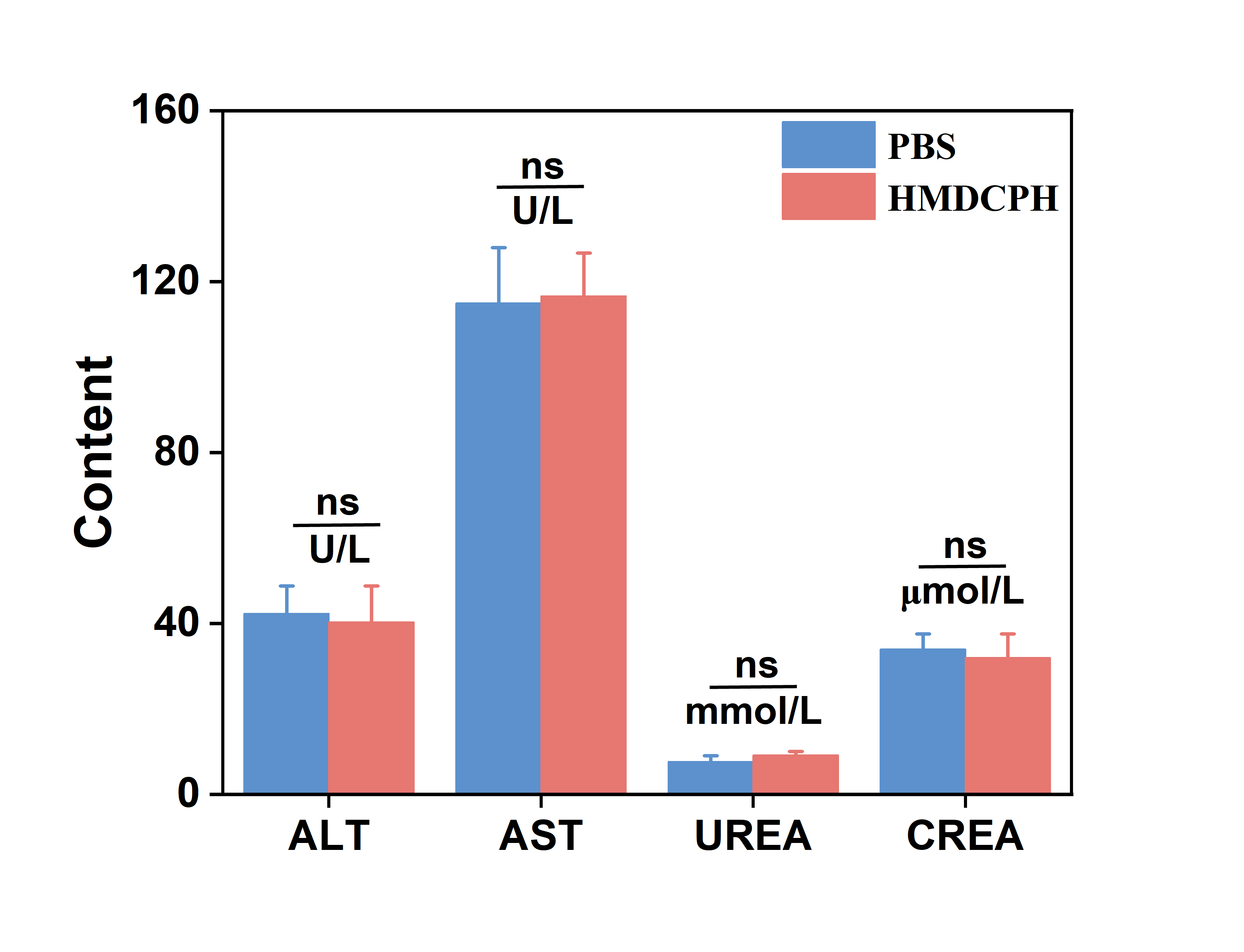


Fig. S11. Blood biochemical parameters of tumor-bearing nude mice from different treatment groups.


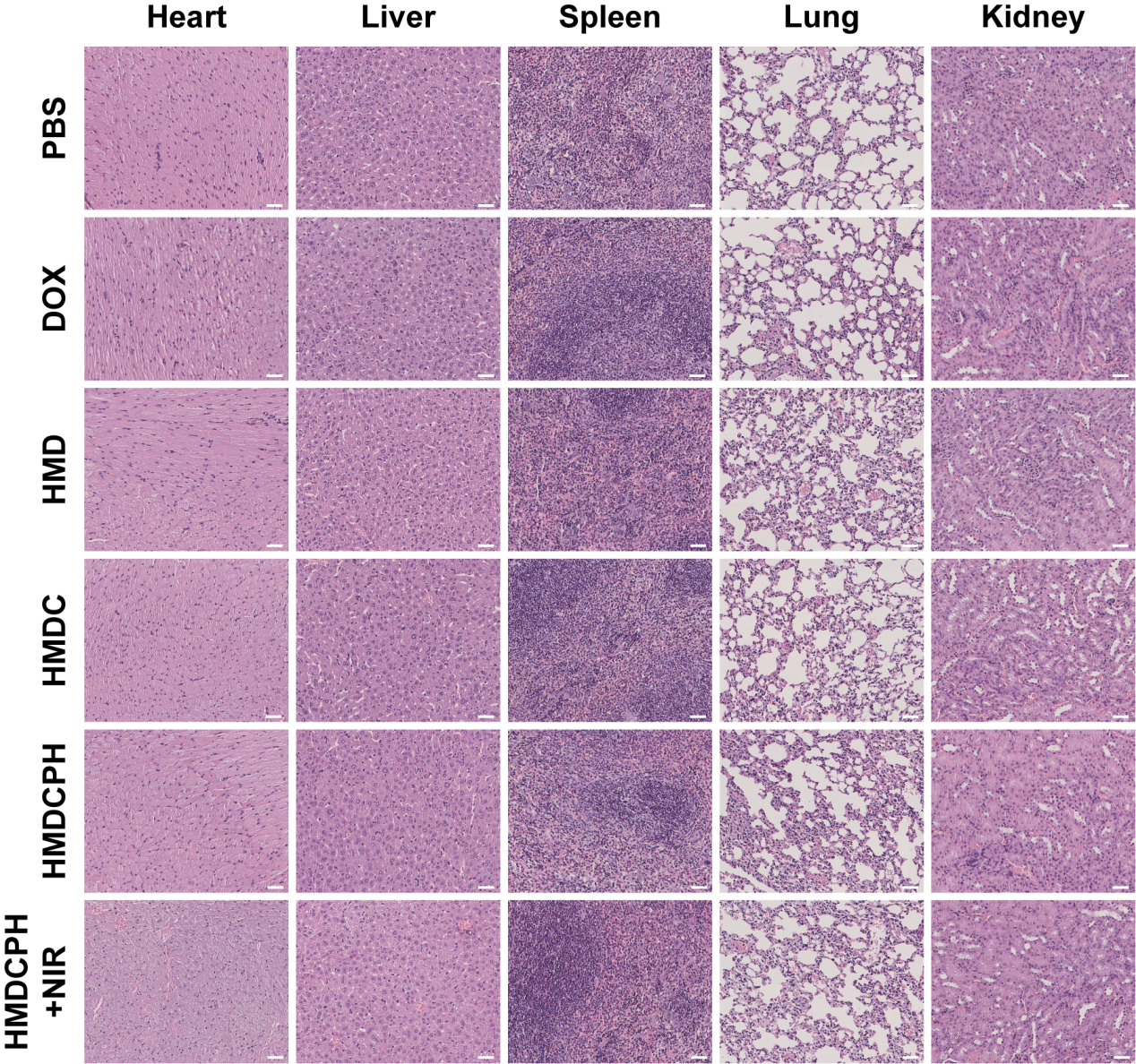


Fig. S12. Representative H&E staining images of major organs from tumor-bearing nude mice in different treatment groups (scale bar: 20 μm).
